# The role of the encapsulated cargo in microcompartment assembly

**DOI:** 10.1371/journal.pcbi.1006351

**Published:** 2018-07-31

**Authors:** Farzaneh Mohajerani, Michael F. Hagan

**Affiliations:** Martin A. Fisher School of Physics, Brandeis University, Waltham, Massachusetts, United States of America; Hebrew University of Jerusalem, ISRAEL

## Abstract

Bacterial microcompartments are large, roughly icosahedral shells that assemble around enzymes and reactants involved in certain metabolic pathways in bacteria. Motivated by microcompartment assembly, we use coarse-grained computational and theoretical modeling to study the factors that control the size and morphology of a protein shell assembling around hundreds to thousands of molecules. We perform dynamical simulations of shell assembly in the presence and absence of cargo over a range of interaction strengths, subunit and cargo stoichiometries, and the shell spontaneous curvature. Depending on these parameters, we find that the presence of a cargo can either increase or decrease the size of a shell relative to its intrinsic spontaneous curvature, as seen in recent experiments. These features are controlled by a balance of kinetic and thermodynamic effects, and the shell size is assembly pathway dependent. We discuss implications of these results for synthetic biology efforts to target new enzymes to microcompartment interiors.

## Author summary

Bacterial microcompartments are protein shells that encase enzymes and reactants to enable bacteria to perform vital reactions, such as breaking down chemicals for energy or converting the products of photosynthesis into sugars. Microcompartments are essential for many bacteria, including human pathogens. Thus, there is great interest in understanding how microcompartment shells assemble around their cargo (the interior enzymes and reactants), and what determines the structure and size of a microcompartment. These questions are difficult to answer with experiments alone, because most intermediates in the assembly process are too short-lived to characterize in experiments. Therefore, this article describes theoretical and computational models for microcompartments, which predict assembly pathways and how the sizes of assembled shells depend on factors such as protein interactions and concentrations. The simulations show that the properties of the cargo are an important factor for determining shell size, and suggest an explanation for recent experimental results showing that cargo can either increase or decrease shell size. In addition to helping to understand the natural behavior of microcompartments, the simulations provide guidance to researchers working to reengineer microcompartments to produce drugs or biofuels.

## Introduction

While it has long been recognized that membrane-bound organelles organize the cytoplasm of eukaryotes, it is now evident that protein-based compartments play a similar role in many organisms. For example, bacterial microcompartments (BMCs) are icosahedral proteinaceous organelles that assemble around enzymes and reactants to compartmentalize certain metabolic pathways [1–10]. BMCs are found in at least 20% of bacterial species [2, 11, 12], where they enable functions such as growth, pathogenesis, and carbon fixation [1, 10, 13–16]. Other protein shells act as compartments in bacteria and archea, such as encapsulins [17] and gas vesicles [17, 18], and even in eukaryotes (*e.g*. vault particles [19]). Understanding the factors that control the assembly of BMCs and other protein-based organelles is a fundamental aspect of cell biology. From a synthetic biology perspective, understanding factors that control packaging of the interior cargo will allow reengineering BMCs as nanocompartments that encapsulate a programmable set of enzymes, to introduce new or improved metabolic pathways into bacteria or other organisms (*e.g*. [10, 20–29])]. More broadly, understanding how the properties of a cargo affect the assembly of its encapsulating container is important for drug delivery and nanomaterials applications.

Despite atomic resolution structures of BMC shell proteins [1, 10, 30, 31], the factors that control the size and morphology of assembled shells remain incompletely understood. BMCs are large and polydisperse (40-600 nm diameter), with a roughly icosahedral protein shell surrounding up to thousands of copies of enzymes [1, 7–9, 30, 32, 33]. For example, the best studied BMC is the carboxysome, which encapsulates RuBisCO and carbonic anhydrase to facilitate carbon fixation in cyanobacteria [1, 30, 32, 34]. BMC shells assemble from multiple paralogous protein species, which respectively form homo-pentameric, homo-hexameric, and pseudo-hexameric (homo-trimeric) oligomers [1, 30, 31]. Sutter *et al*. [31] recently obtained an atomic-resolution structure of a complete BMC shell in a recombinant system that assembles small (40 nm) empty shells (containing no cargo). The structure follows the geometric principles of icosahedral virus capsids, exhibiting *T* = 9 icosahedral symmetry in the Caspar-Klug nomenclature [35, 36] (meaning there are 9 proteins in the asymmetric unit). The pentamers, hexamers, and pseudo-hexamers occupy different local symmetry environments.

Although the Sutter *et al*. [31] structure marks a major advance in understanding microcompartment architectures, it is uncertain how this construction principle extends to natural microcompartments, which are large (100-600 nm), polydisperse, and lack perfect icosahedral symmetry. Moreover, the effect of cargo on BMC shell size is hard to interpret from experiments. In some BMC systems, empty shells are smaller and more monodisperse than full shells [23, 28, 31, 37], whereas in other systems empty shells are larger than full ones [38]. Thus, the cargo may increase or decrease shell size.

The encapsulated cargo can also affect BMC assembly pathways. Microscopy experiments showed that *β*-carboxysomes (which encapsulate form 1B RuBisCO) undergo two-step assembly: first the enzymes coalesce into a ‘procarboxysome’, then shells assemble on and bud from the procarboxysome [39, 40]. In contrast, electron micrographs suggest that *α*-carboxysomes (another type of carboxysome that encapsulates form 1A RuBisCO) assemble in one step, with simultaneous shell assembly and cargo coalescence [33, 41]. Our recent computational study [42] suggested that the assembly pathway depends on the affinity between cargo molecules. However, that study was restricted to a single shell size, and thus could not investigate correlations between assembly pathway and shell size.

Numerous modeling studies have identified factors controlling the thermodynamic stability [43–45] or dynamical formation [46–54] of empty icosahedral shells with different sizes. For example, Wagner and Zandi showed that icosahedral shells can form when subunits sequentially and irreversibly add to a growing shell at positions which globally minimize the elastic energy, with the preferred shell size determined by the interplay of elastic moduli and protein spontaneous curvature. Several studies have also investigated the effect of templating by an encapsulated nanoparticle or RNA molecule on preferred shell size [50, 55–57]. However, the many-molecule cargo of a microcompartment is topologically different from a nucleic acid or nanoparticle, and does not template for a specific curvature or shell size.

Rotskoff and Geissler recently proposed that microcompartment size is determined by kinetic effects arising from templating by the cargo [58]. Using an elegant Monte Carlo (MC) algorithm they showed that proteins without spontaneous curvature, which form polydisperse aggregates in the absence of cargo, can form kinetically trapped closed shells around a cargo globule. However, there are reasons to question the universality of this mechanism for microcompartment size control. Firstly, several recombinant BMC systems form small, monodisperse empty shells [23, 28, 31, 37], suggesting that the shell proteins have a non-zero spontaneous curvature even without cargo templating. Secondly, when Cameron *et al*. [39] overexpressed RuBisCO to form ‘supersized’ procarboxysomes, carboxysome shells encapsulated only part of the complex, suggesting that there is a maximum radius of curvature that can be accommodated by the shell proteins. Thirdly, the kinetic mechanism is restricted to systems in which rates of shell association vastly exceed cargo coalescence rates, a condition which may not apply in biological microcompartment systems. Thus, despite this and other recent simulation studies of microcompartments [42, 58, 59], the factors which control BMC size and amount of encapsulated cargo remain unclear.

In this article we use equilibrium calculations and Brownian dynamics (BD) simulations on a minimal model to identify the factors that control the size of a microcompartment shell. Although computationally more expensive than the MC algorithm of Ref. [58], BD better describes cooperative cargo-shell motions and thus allows for any type of assembly pathway. Using this capability, we explore the effect of cargo on shell size and morphology over a range of parameters leading to one-step or two-step assembly pathways. To understand the interplay between shell curvature and cargo templating, we consider two limits of shell protein interaction geometries: zero spontaneous curvature and high spontaneous curvature, which respectively form flat sheets or small icosahedral shells in the absence of cargo.

Our calculations find that the presence of cargo can increase or decrease shell size, depending on the stoichiometry of cargo and shell proteins, and the protein spontaneous curvature. For shell proteins with high spontaneous curvature, we observe a strong correlation between assembly pathway and shell size, with two-step assembly leading to larger shells than single-step pathways or empty shell assembly. This result is consistent with the fact that *β*-carboxysomes tend to be larger than *α*-carboxysomes. For shell proteins with zero spontaneous curvature, we find that introducing cargo can result in a well-defined shell size through several mechanisms, including the kinetic mechanism of Ref. [58] and the ‘finite-pool’ effect due to a limited number of cargo particles available within the cell. However, spontaneous curvature of the shell proteins allows for robust shell formation over a wider range of parameter space.

## Materials and methods

### Computational model

#### Shell subunits

**Fig 1:**
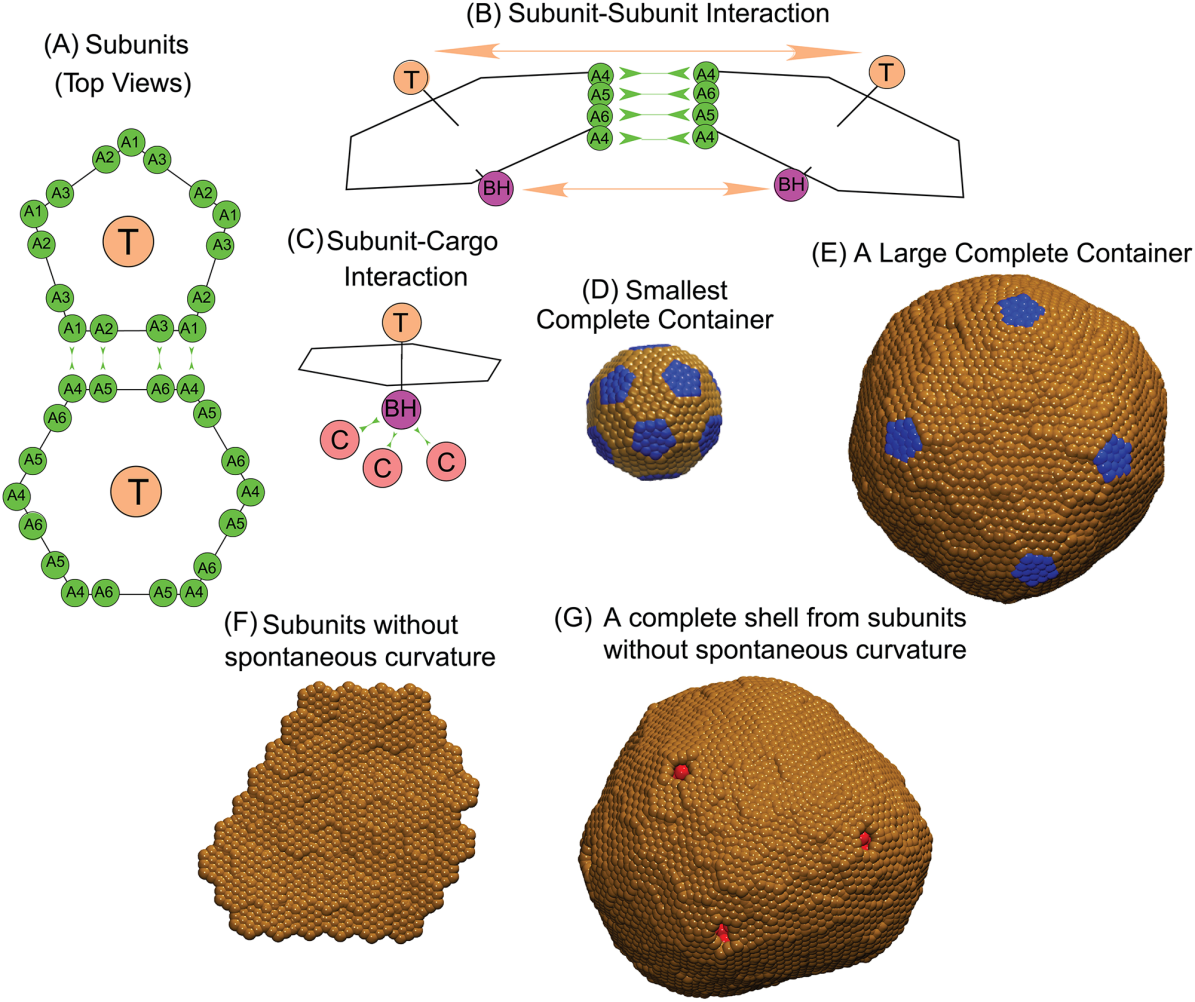
Description of the model. **(A)** Each shell subunit contains ‘Attractors’ (green circles) on the perimeter, a ‘Top’ (tan circle, ‘T’) in the center above the plane, and a ‘Bottom’ (purple circle, ‘BH’ and ‘BP’ below the planes of the hexamer and the pentamer respectively). **(B)** Interactions between Attractors drive subunit binding, while Top-Top and Bottom-Bottom repulsions control the subunit-subunit angle and the shell bending modulus *κ*_s_. Attractions are indicated by green arrows in (A) for the pentamer-hexamer interface and in (B) for the hexamer-hexamer interface. **(C)** Only hexamer Bottom psuedoatoms ‘BH’ bind cargo molecules (terra cotta circles, ‘C’). Excluder atoms (blue and brown pseudoatoms in **(D)**) placed in the plane of the ‘Top’ experience excluded volume interactions with the cargo. **(D)** The positions of excluder atoms in the preferred shell geometry for subunits with spontaneous curvature, a truncated icosahedron with 12 pentamers (blue) and 20 hexamers (brown). **(E)** Example of a shell that is larger than the preferred subunit geometry. **(F)** Subunits without spontaneous cuvature. **(G)** Example of hexamers without spontaneous curvature assembled around cargo (red).

BMC shells assemble from pentameric (BMC-P), hexameric (BMC-H), and pseudo-hexameric (trimeric, BMC-T) protein oligomers (*e.g*. Fig. 3A in Ref. [31] and Refs. [1, 10, 30]). Experimental evidence suggests these oligomers are the basic assembly units, meaning that smaller complexes do not contribute significantly to the assembly process [30, 60]. Although a recent atomic-resolution structure of synthetic BMC shells identifies specific roles for hexamers and pseudo-hexamer species [31], it is unclear how these roles extend to larger shells. Therefore, for simplicity our model considers two basic assembly subunits, pentamers and hexamers, with the latter fulfilling the roles of both hexamers and pseudo-hexamers. We consider a minimal model which captures the directional interactions and excluded volume shape of subunits inferred from the recent structure [31], and the fact that a closed shell is impermeable to cargo particles. Our model builds on previous models for virus assembly [51, 61–66] and our recent model for the assembly around a fluid cargo [42]. However, while that model was specific to *T* = 3 shells (containing 12 pentamers and 20 hexamers in a truncated icosahedron geometry), we have extended the model to describe shells of any size (see Fig 1). A survey of other models which have been used for icosahedral shells can be found in Refs. [67–69].

#### Shell-shell interactions

Interactions between edges of BMC shell proteins are primarily driven by shape complementarity and hydrophobic interactions [31]. To mimic these short-ranged directionally specific interactions, each model subunit contains ‘Attractors’ on its perimeter that mediate shell-shell attractions. Complementary Attractors on nearby subunits have short-range interactions (modeled by a Morse potential, Eq. (S1.4) in S1 Text). Attractors which are not complementary do not interact. The arrangement of Attractors on subunit edges is shown in Fig 1, with pairs of complementary Attractors indicated by green double-headed arrows. In the previous model [42] different hexamer edges interacted with either hexamers or pentamers, which made the model specific to the smallest possible shell, (a *T* = 3 structure, Fig 1D). In this work, we allow for any shell geometry by making the hexamers six-fold symmetric, with each edge attracted to any edge on a nearby hexamer or pentamer. However, because there is no experimental evidence of pentamer proteins (BMC-P) forming higher order assemblies (except non-specific aggregates) in the absence of hexamer proteins [70], we do not consider attractive interactions between pairs of pentamers.

The parameters *ε*_HH_ and *ε*_PH_ scale the well-depths of the Morse potential between complementary Attractors for hexamer-hexamer and hexamer-pentamer interactions, and are thus the parameters that control the shell-shell binding affinity. Further model details are in section S1 Text.

To control the shell spontaneous curvature and bending modulus, each subunit contains a ‘Top’ (type ‘TP’ and ‘TH’ for pentamers and hexamers respectively) pseudoatom above the plane of Attractors, and a ‘Bottom’ pseudoatom (Types ‘BP’ and ‘BH’ for pentamers and hexamers respectively) below the Attractor plane. There are repulsive interactions (cutoff Lennard-Jones interactions, Eq. (S1.3)) between Top-Top, Bottom-Bottom, and Top-Bottom pairs of pseudoatoms on nearby subunits. The relative sizes of the Top and Bottom pseudoatoms set the preferred subunit-subunit binding angle (and thus the spontaneous curvature), while the interaction strength (controlled by the well-depth parameter *ε*_angle_) controls the shell bending modulus *κ*_s_. We performed simulations of assembled shells to measure the relationship *κ*_s_(*ε*_angle_), as described in section S1 Text. The Top-Bottom interaction ensures that subunits do not bind in inverted orientations [51]. For subunits with no spontaneous curvature, we have extended simulations into the limit of small *κ*_s_ values, for which the Top-Top and Bottom-Bottom repulsive interactions are insufficient to avoid partial subunit overlap. Therefore we have added an additional pseudoatom for subunits with no spontaneous curvature, a middle pseudoatom ‘M’ placed in the center of the subunit in the plane of the attractors. The addition of ‘M’ pseudoatoms does not affect behaviors for *ε*_angle_ ≥ 0.5, and prevents overlaps below this range.

#### Shell-cargo interactions

Attractive interactions between hexamers and cargo are modeled by a a Morse potential with well-depth parameter *ε*_SC_ between cargo particles (type ‘C’) and Bottom pseudoatoms on hexamers (type ‘BH’). These interactions represent shell-cargo attractions mediated by ‘encapsulation peptides’ in BMCs [38, 39, 71–73]. Because there is no experimental evidence that such encapsulation peptides interact with pentamers, in our model ‘BP’ pseudoatoms do not interact with cargo particles. We also add a layer of ‘Excluders’ in the plane of the ‘Top’ pseudoatoms, which represent shell-cargo excluded volume interactions. Since the shell-shell interaction geometries are already controlled by the Attractor, Top, and Bottom pseudoatoms, we do not consider Excluder-Excluder interactions.

#### Cargo

In carboxysome systems, attractions between RuBisCo particles are mediated by auxiliary proteins (*e.g*. the protein CcmM in *β*−carboxysomes [39]). In refs [39, 40] these interactions were shown to drive coalescence of RuBisCO prior to budding of *β*−carboxysomes assembled shells. Similarly, experiments and theory [74] support that protein-mediated phase separation of RuBisCO occurs in the pyrenoid, a dense complex of RuBisCO responsible for carbon fixing in plants. Since the complete phase diagram of RuBisCO and its auxiliary proteins is not known, we capture the possibility of cargo phase separation in the simplest manner possible by representing the cargo as spherical particles that interact via an attractive Lennard-Jones (LJ) potential, with well-depth *ε*_CC_. Perlmutter *et al*. [42] found that a more realistic, anisotropic model of the RuBisCO octomer holoenzyme did not qualitatively change assembly behaviors in comparison to spherical cargo particles [42].

The phase diagram of LJ particles contains regions of vapor, liquid, and solid and coexistence regimes [75]. In this work we consider only one cargo density $0.0095/\sigma_{0}^{3}$, for which vapor-liquid coexistence begins at *ε*_CC_ = 1.5 and the liquid-solid transition occurs at *ε*_CC_ = 2.2. Note that vapor-liquid coexistence in our finite system requires slightly stronger interactions than in the thermodynamic limit.

This model captures the excluded volume shape of subunits and their general binding modes observed in the microcompartment shell crystal structure [31]. Further refinements of the model are possible based on that structure, including an explicit representation of pseudo-hexamers and incorporating different preferred binding angles for pentamer-hexamer, hexamer-hexamer and hexamer-pseudo-hexamer interactions. It would be interesting to consider continued input of cargo or shell subunits into the system during assembly. Theoretical studies have suggested that a dynamical supply of subunits can affect the behavior of capsid assembly [76–80].

### Simulations

We simulated assembly dynamics using the Langevin dynamics algorithm in HOOMD (which uses GPUs to efficiently simulate dynamics [81]), and periodic boundary conditions to represent a bulk system. The subunits are modeled as rigid bodies [82]. Each simulation was performed in the NVT ensemble, using a set of fundamental units [83] with *σ*_0_ defined as the circumradius of the pentagonal subunit (the cargo diameter is also set to *σ*_0_), and energies given in units of the thermal energy, *k*_B_*T*. The simulation time step was 0.005 in dimensionless time units, and we performed 3 × 10^6^ timesteps in each simulation unless mentioned otherwise.

*Initial conditions*. We considered two types of initial conditions. Except where stated otherwise, simulations started from the ‘homogeneous’ initial condition, in which subunits and (if present) cargo were initialized with random positions and orientations, excluding high-energy overlaps. In the ‘pre-equilibrated globule’ initial condition, we first initialized cargo particles with random positions (excluding high-energy overlaps), and performed 10^5^ simulation timesteps to equilibrate the cargo particles. Shell subunits were then added to the simulation box with random positions and orientations, excluding high-energy overlaps.

*Systems*. We simulated several systems as follows. For shell subunits with spontaneous curvature we set pentamer-hexamer and hexamer-hexamer angles consistent with the *T* = 3 geometry (see Estimating the shell bending modulus in section S2 Text), and we set *ε*_angle_ = 0.5. We first performed a set of empty-shell assembly simulations, with 360 hexamers, and varying number of pentamers, in a cubic box with side length 60*σ*_0_, with *ε*_HH_ = 2.6*k*_B_*T* (the smallest interaction strength for which nucleation occurred). These simulations were performed for 10^7^ timesteps to obtain sufficient statistics at low pentamer concentrations despite nucleation being rare.

For cargo encapsulation by subunits with spontaneous curvature, we simulated 2060 cargo particles, 180 pentamers, and 360 hexamers in a cubic box with side length 60*σ*_0_. Other parameters were the same as for the empty-shell simulations, except that we varied *ε*_PH_, *ε*_SC_, and *ε*_SC_ as described in the main text. All simulations with spontaneous curvature used *ε*_PH_ ≥ 1.3*ε*_HH_ to ensure that the shells with the *T* = 3 geometry (or asymmetric shells with similar sizes) were favored in the absence of cargo. We note that our results generalize to other ranges of shell interaction parameters, but this choice distinguishes effects due to cargo from those due to changes in the inherent preferred shell geometry. Simulations with strong cargo-cargo and cargo-shell interactions (*ε*_CC_ ≥ 1.55 and *ε*_SC_ < 8.75) required a long timescale for pentamers to fill pentameric vacancies in the hexamer shell (discussed in Results). To observe pentamer adsorption, these simulations were run for up to 9 × 10^6^ simulation timesteps.

For simulations of ‘flat’ subunits (with no spontaneous curvature), we considered a range of system sizes at fixed steady state cargo chemical potential, with the number of cargo particles varying from 409 to 3275, and the box side length varying from 35*σ*_0_ to 70*σ*_0_. Since these were NVT simulations, we ensured that the final hexamer chemical potential was the same at each system size by setting the number of hexamers so that the concentration of free hexamers remaining after assembly of a complete shell was constant (10^−3^ subunits/$\sigma_{0}^{3}$). The resulting number of hexamers varied from 109 to 581 in boxes with side lengths 35*σ*_0_ to 70*σ*_0_. The assembly outcomes were unchanged if instead we kept the total hexamer subunit concentration the same across all simulations. For each of these system sizes we performed simulations over a range of *ε*_angle_ to identify the maximum value of *κ*_s_ at which assembly of a complete shell could occur. Simulations were stopped upon completion of a shell or after the maximum simulation time *t*_max_ with *t*_max_ = 3 × 10^6^ timesteps for boxes with side length ≤ 55*σ*_0_ and *t*_max_ = 8 × 10^6^ for boxes with side length ≥ 55*σ*_0_. The maximum simulation time was increased for large system sizes because the minimum time required for assembly of a complete shell increases linearly with the shell size [84].

To estimate the relationship between the shell bending modulus *κ*_s_ and the parameter *ε*_angle_ we performed additional simulations, in which we measured the total interaction energy of completely assembled shells as a function of *ε*_angle_ (see ‘Estimating the shell bending modulus’ in section S2 Text).

*Sample sizes*. For simulations of shells with spontaneous curvature, we performed a minimum of 10 independent trials at each parameter set. To enable satisfactory statistics on shell size and morphology for parameter sets that result in at most one complete shell in the simulation box 3, we performed additional trials such that at least 10 complete shells were simulated. For flat subunits (Fig 1F and 1G), we identified the maximum *ε*_angle_ for which a complete shell forms at each system size as follows. We first performed independent simulations over a range of *ε*_angle_ values, separated by increments in *ε*_angle_ of 0.02 for systems with box side length ≤ 55*σ*_0_, and increments of 0.05 for systems with side length ≥ 55*σ*_0_. We performed 10 independent trials at each value of *ε*_angle_. For the largest value of *ε*_angle_ at which at least one of these trials resulted in a complete shell, we then performed 10 additional trials to obtain a more accurate estimate of the shell bending modulus *κ*_s_ at the maximum *ε*_angle_.

## Results and discussion

To simulate the dynamics of microcompartment assembly, we build on the model developed by Perlmutter *et al*. [42], which allowed only a single energy minimum shell geometry, corresponding to a *T* = 3 icosahedral shell containing 12 pentamers and 20 hexamers. We have now extended the model to allow for closed shells of any size. Based on AFM experiments showing that BMC shell facets assemble from pre-formed hexamers [60], and the fact that carboxysome major shell proteins crystallize as pentamers and hexamers [30], our model considers pentamers and hexamers as the basic assembly units. These are modeled as rigid bodies with short-range attractions along their edges, which drive hexamer-hexamer and hexamer-pentamer association. Repulsive subunit-subunit interactions control the preferred angle of subunit-subunit interactions, which sets the shell protein spontaneous curvature (Fig 1A and 1B). To minimize the number of model parameters, we do not explicitly consider pseudo-hexamers; thus, the model hexamers play the role of both hexamers and pseudo-hexamers.

We particularly focus on carboxysomes, for which the most experimental evidence is available, although our model is sufficiently general that results are relevant to other microcompartment systems. In carboxysomes, interactions between the RuBisCO cargo and shell proteins are mediated by non-shell proteins containing ‘encapsulation peptides’ [39, 41, 71, 85–88]. For simplicity we model these interactions as direct-pair attractions between model cargo particles and shell subunits. Because there is no evidence that encapsulation peptides interact with pentamers, in our model the cargo only interacts with hexamers. Further details of the model and a thermodynamic analysis are given in section Materials and methods and section S2 Text.

There are numerous parameters which can affect shell size, including the interaction strengths among the various species of cargo and shell subunits, shell protein spontaneous curvature and bending modulus, and the concentration of each species. To facilitate interpretation of results from this vast parameter space, we focus our simulations on two extreme limits. In the first limit, we consider shell subunits with a spontaneous curvature that favors assembly of the smallest icosahedral shell, the *T* = 3 structure with 12 pentamers and 20 hexamers (Fig 1D). In the second limit we consider a system containing only hexamer subunits with no preferred curvature, which form flat sheets without cargo (Fig 1F).

### Cargo increases the size of shells with high spontaneous curvature

**Fig 2:**
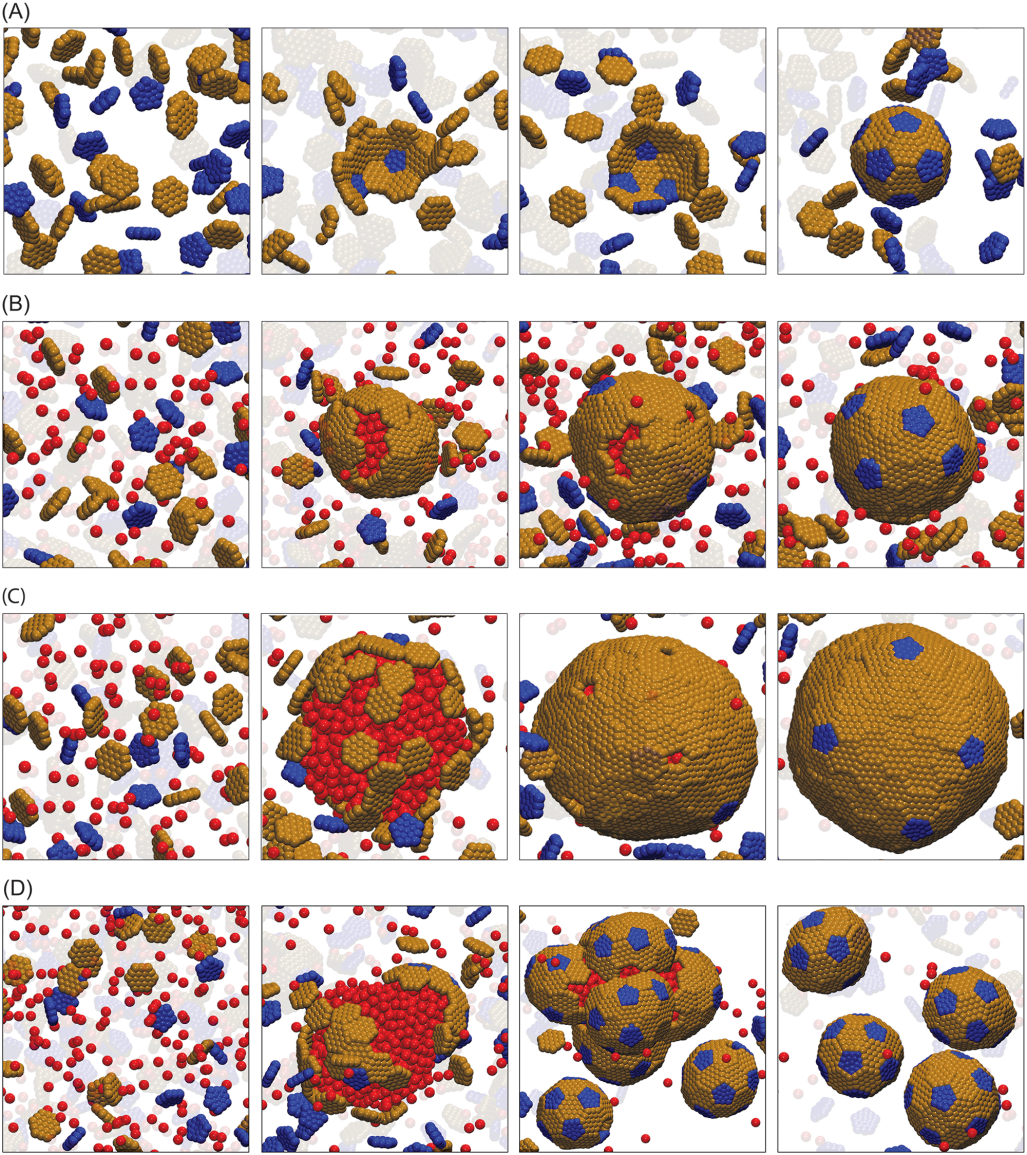
Snapshots from assembly trajectories of subunits with *T* = 3 preferred curvature. **(A)** Small *T* = 3 shells (20 hexamers, 12 petamers) assembled without cargo at *ε*_HH_ = 2.6 and pentamer/hexamer stoichiometric ratio *ρ*_p_/*ρ*_h_ = 0.5. Notice that the intermediate in the third frame contains a hexamer where a pentamer is required for icosahedral symmetry. This hexamer eventually dissociates. **(B)** One-step assembly with moderate cargo-cargo interaction strength, *ε*_CC_ = 1.5. A small nucleus of cargo and hexamer subunits forms, followed by simultaneous cargo coalescence, shell growth, and finally filling in of defects by pentamers subunits. The final structure has 68 hexamers, 12 pentamers, and 408 encapsulated cargo particles. Other parameters are hexamer-hexamer affinity *ε*_HH_ = 1.8, ratio of pentamer/hexamer affinity *ε*_PH_/*ε*_HH_ = 1.3, and shell-cargo affinity *ε*_SC_ = 8.75, and *ρ*_p_/*ρ*_h_ = 0.5. **(C)** Two-step assembly pathway for strong cargo-cargo affinity *ε*_CC_ = 1.65. Rapid cargo coalescence is followed by adsorption and assembly of shell subunits. The final structure has 167 hexamers, 12 pentamers, and 1520 encapsulated cargo particles. Other parameters are *ε*_HH_ = 1.8, *ε*_SC_ = 8.5, and *ρ*_p_/*ρ*_h_ = 0.5. **(D)** Assembly and budding of shells from a cargo globule, for high pentamer/hexamer affinity ratio *ε*_PH_/*ε*_HH_ = 2.0. Other parameters are *ε*_CC_ = 1.65, *ε*_HH_ = 1.8, *ε*_SC_ = 8.5 and *ρ*_p_/*ρ*_h_ = 0.8. (We report energies in units of *k*_B_*T* throughout this article.) The shell bending modulus for all panels is *κ*_s_ = 10*k*_B_*T*. Animations corresponding to these trajectories are provided in S1, S2 and S3 Videos.

We begin by considering shells with *T* = 3 spontaneous curvature (Fig 1D). To isolate the effects of cargo on shell size, we consider shell-shell interaction parameters which favor pentamer insertion (setting the ratio of pentamer-hexamer and hexamer-hexamer affinities *ε*_PH_/*ε*_HH_ ≥ 1.3) so that assembly without cargo results in primarily *T* = 3 empty shells for our ratio of pentamer to hexamer concentrations, *ρ*_p_/*ρ*_h_ = 0.5, and results in shells close in size to the T = 3 geometry at all of the stoichiometries we consider here. A typical assembly trajectory without cargo is shown in Fig 2A. When simulating assembly around cargo, we set the hexamer-hexamer affinity *ε*_HH_ ≤ 2.2 (while maintaining *ε*_PH_/*ε*_HH_ ≥ 1.3) so that assembly occurs only in the presence of cargo, and we vary cargo-cargo *ε*_CC_ and cargo-shell *ε*_SC_ interaction strengths. Throughout this article, all energy values are given in units of the thermal energy, *k*_B_*T*. Except where mentioned otherwise, values of our simulation shell bending modulus *κ*_s_ fall within the range estimated for *β*−carboxysomes from AFM nanoindention experiments *κ*_s_ ∈ [1, 25]*k*_B_*T* (see Ref. [89] and section ‘Determination of parameter values’ in S2 Text; simulations with shell spontaneous curvature use *κ*_s_ = 10 − 16*k*_B_*T*.

#### Assembly pathways

Consistent with previous simulations of *T* = 3-specific shells [42], we find that assembly proceeds by one-step or two-step pathways, with the type of pathway primarily determined by the strength of cargo-cargo interactions. For *ε*_CC_ ≲ 1.5 (Fig 2B), the cargo lies at or below the border of phase coexistence, and there is a large barrier for cargo coalescence. However, a fluctuation in the local density of hexamers allows nucleation of a small cargo globule and shell cluster, after which cargo condensation, shell subunit adsorption and assembly occur simultaneously. On the other hand, for *ε*_CC_ ≳ 1.55*k*_B_*T* (Fig 2C) a cargo globule coalesces rapidly. Hexamers then adsorb onto the cargo globule in a disordered manner, followed by reorganization and assembly. Since pentamers are not directly attracted to the cargo, they are mostly excluded for the pentamer/hexamer affinity ratio, *ε*_PH_/*ε*_HH_ = 1.3, considered in Fig 2C. However, the hexamers cannot form a closed surface around the globule since the spherical topology requires 12 five-fold defects [90]. Interestingly, for moderate interaction strengths we find that shells satisfy this requirement by forming exactly 12 pentamer-sized vacancies in the shell, which are gradually filled in by pentamers. Increasing the pentamer/hexamer affinity ratio to *ε*_PH_/*ε*_HH_ = 2 (Fig 2D) allows pentamers to rapidly bind to adsorbed hexamers, creating additional shell curvature and thus driving the budding of small shells containing part of the globule in their interior.

The shells assembled around cargo are larger and lack the perfect icosahedral symmetry of the intrinsic preferred shell geometry (*T* = 3, 20 hexamers). Despite the lack of symmetry, most shells are closed, meaning that every hexamer and pentamer subunit interacts with respectively six and five neighboring subunits. The yield and fraction of complete shells are shown in S1 and S2 Figs. Once a complete shell forms with or without cargo, it is stable on assembly timescales even under infinite dilution of subunits. This hysteresis between assembly and disassembly is consistent with previous experimental and theoretical studies of virus assembly [67, 91–94], and occurs because removal of the first subunit from a complete shell breaks multiple contacts thus incurring a large activation barrier.

S3 Fig shows the Steinhardt icosahedral order parameter as a function of shell size along with snapshots of typical shells. We observe that the degree of icosahedral symmetry increases with shell size, and is correlated to the assembly pathway. Small shells that assemble by one-step pathways (with ∼ 50 subunits) are clearly asymmetric, corresponding neither to icosahedral symmetry nor other symmetric low-energy minimum arrangements expected for shells in this size range [95], whereas large shells are nearly (though not perfectly) icosahedral. The lack of perfect symmetry likely arises because the hexamers form an elastic sheet, within which shell reorganization and defect diffusion are slow in comparison to assembly timescales. Based on analysis of assembly trajectories, we speculate that the higher degree of symmetry for large shells reflects the fact that pentamers are incorporated near the end of two-step pathways (filling in pentamer-sized vacancies) whereas pentamers incorporate early in one-step pathways. Because rearranging a pentamer within a shell requires breaking more bonds than does a vacancy, pentamer rearrangement is slower than vacancy diffusion.

#### Shell size depends on interaction strengths, subunit stoichiometry, and initial conditions

**Fig 3:**
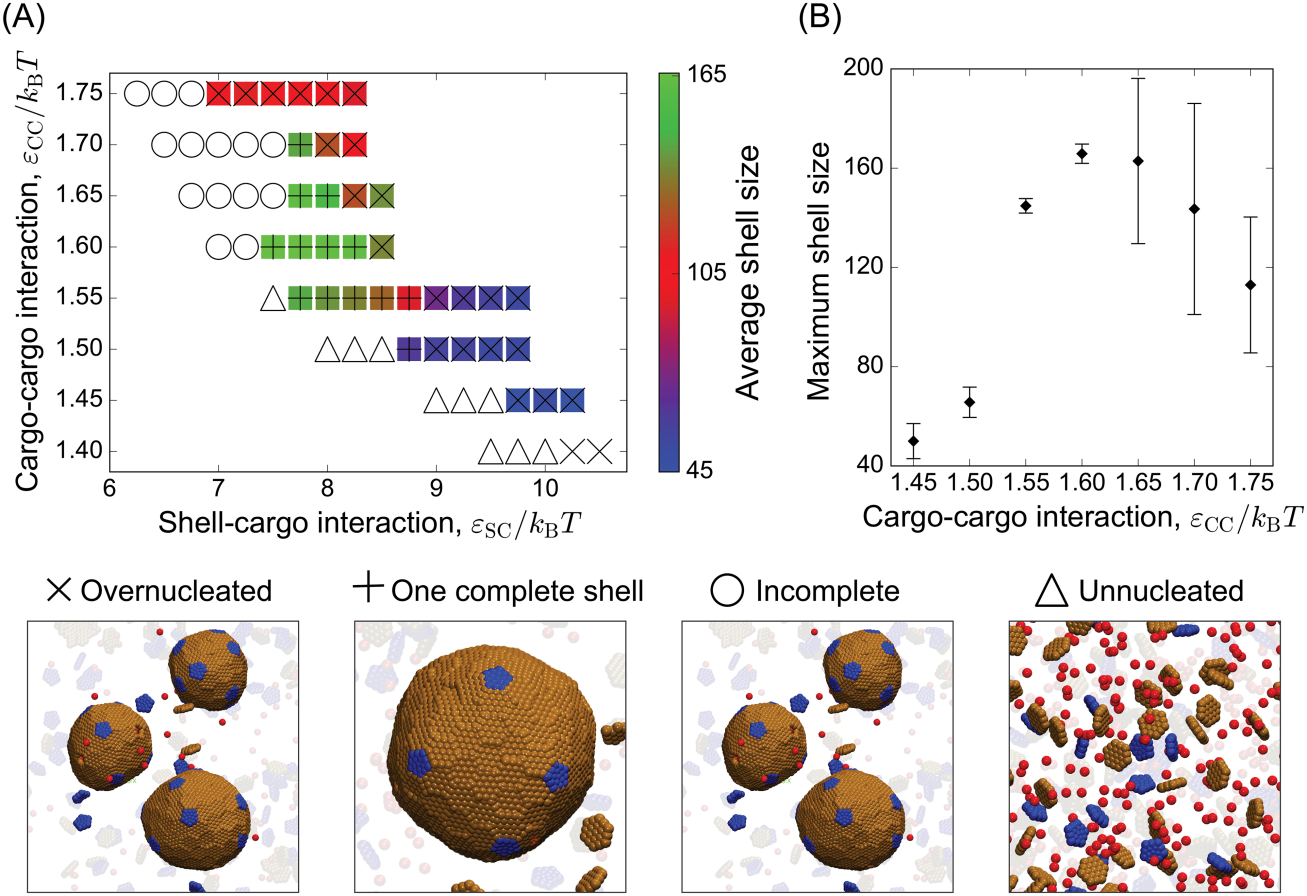
Dependence of the mean shell size and most probable morphology on the cargo-cargo and subunit-cargo affinities (*ε*_CC_ & *ε*_SC_). **(A)** The mean shell size (number of hexamers + 12 pentamers) is indicated by the color bar, and the predominant morphology is indicated by symbols, with a snapshot corresponding to each morphology shown on the right. **(B)** The mean shell size maximized over *ε*_SC_ is shown as a function of *ε*_CC_. Other parameters in (A) and (B) are *ε*_HH_ = 1.8, *ρ*_p_/*ρ*_h_ = 0.5, *ε*_PH_/*ε*_HH_ = 1.3, and *κ*_s_ = 10*k*_B_*T*.

Fig 3A shows the mean size and predominant assembly morphology as a function of cargo-cargo and cargo-shell interaction strengths. Over a wide range of parameter space, shell sizes are larger than the *T* = 3 size formed by empty shells (32 subunits), demonstrating that the cargo can robustly increase shell size. As the shell-cargo interaction is increased within the two-step regime (*ε*_CC_ ≳ 1.55), there is a sequence of predominant assembly outcomes. Weak interactions lead to a disordered layer of shell subunits on the cargo globule, moderate interactions result in one complete shell, and overly strong interactions drive multiple nucleation events throughout the system. This over-nucleation decreases the mean shell size since the system becomes depleted of cargo and shell subunits. The one-step regime exhibits a similar sequence, except that instead of a disordered globule there is no nucleation for weak shell-cargo interactions.

*Pathway dependence*. A striking feature of Fig 2B and 2C is that the two-step assembly pathway leads to much larger shells than the one-step pathway, increasing the number of encapsulated cargo particles by more than a factor of five. We observe a similar correlation between shell size and assembly pathway across the range of simulated parameters. To emphasize the effect of cargo-cargo interactions on shell size, Fig 3B shows the maximum shell size obtained as a function of *ε*_CC_ (maximized over *ε*_SC_). We see a dramatic increase in shell size as the cargo-cargo interactions increase beyond *ε*_CC_ = 1.5, when the system transitions to two-step assembly pathways. The maximum shell size eventually decreases for *ε*_CC_ ≳ 1.65 due to over-nucleation.

**Fig 4:**
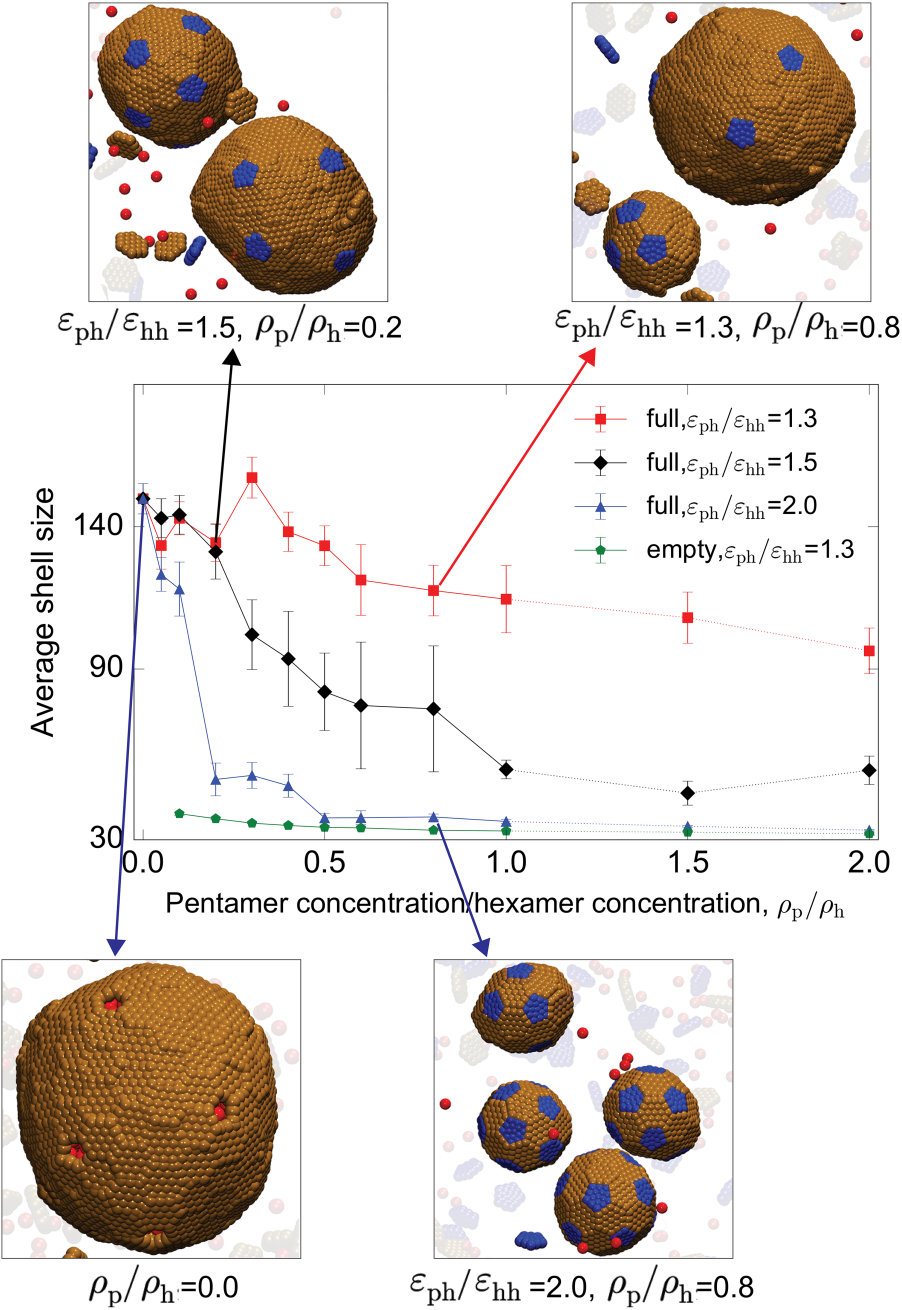
Dependence of shell size on the driving force for pentamer addition. The mean shell size (number of hexamers + 12 pentamers) is shown as a function of the pentamer/hexamer stoichiometry ratio *ρ*_p_/*ρ*_h_ for indicated values of the pentamer/hexamer affinity ratio *ε*_PH_/*ε*_HH_ for simulations with cargo. Results from empty shell simulations are also shown for *ε*_PH_/*ε*_HH_ = 1.3. Snapshots of typical assembly morphologies for indicated parameter values are shown around the plot. In these simulations the hexamer concentration, hexamer-hexamer affinity, and hexamer-shell affinity, and bending modulus were fixed at $\rho_{\text{h}}=1.7\times 10^{-3}/\sigma_{0}^{3}$, *ε*_HH_ = 1.8, *ε*_CC_ = 1.65, *ε*_SC_ = 8.5, and *κ*_s_ = 10*k*_B_*T*.

*Dependence on shell subunit stoichiometry*. To determine the effects of shell subunit stoichiometry, we performed simulations with varying concentrations *ρ*_p_ of pentamers subunits at fixed hexamer concentration. As shown in Fig 4, increasing the pentamer concentration uniformly decreases the shell size. Since only 12 pentamers are required for a closed shell, increasing their chemical potential favors increased pentamer insertion and thus smaller total shell sizes. The effect depends on the pentamer-hexamer affinity; for the moderate pentamer-hexamer interactions considered above (*ε*_PH_ = 1.3*ε*_HH_), we observe a modest decrease in shell size of about 50% with increasing pentamer concentration. In contrast, for strong pentamer-hexamer interactions (*ε*_PH_ = 2*ε*_HH_), even small concentrations of pentamers lead to rapid pentamer insertion and shells that are close in size to the minimum *T* = 3 geometry. At low pentamer stoichiometries we observe very large shells containing approximately 140 subunits; the shell size saturates because it is limited by the droplet size and multi-nucleation events that occur for these relatively strong cargo-cargo and cargo-shell interactions (*ε*_CC_ = 1.65 and *ε*_SC_ = 8.5). In comparison, empty shells with *ρ*_p_/*ρ*_h_ = 0.1 and *ε*_PH_ = 1.3 have a mean size of 39 subunits.

*Kinetics vs. thermodynamics*. Our trajectories start from an out-of-equilibrium condition of unassembled subunits, and reorganization of complete shells is slow in comparison to assembly timescales. Therefore the ensemble of shells that we observe in finite-time simulations can depend on both kinetic and thermodynamic effects. We performed several analyses to assess the relative importance of kinetics and thermodynamics.

First, we investigated whether assembly morphologies depend on initial configurations. For notational clarity, we will refer to the initial condition for simulations described so far, in which shell subunits and cargo start from random positions, as the ‘homogeneous’ initial condition. We performed a second set of simulations started from a ‘pre-equilibrated globule’ initial condition, in which the cargo particles were allowed to completely phase separate before introduction of the shell subunits (see section Materials and methods). When the cargo is below phase coexistence (*ε*_CC_ < 1.5 at the simulated cargo concentration) the two initial conditions produce identical results. Above phase coexistence the pre-equilibrated globule leads to larger globule sizes in comparison to the homogeneous initial condition, since shell assembly tends to arrest globule coalescence. Correspondingly, the pre-equilibrated globule initial condition produces larger shells than the homogeneous initial condition (S4 and S5 Figs). This effect is most significant at the boundary of phase coexistence (*ε*_CC_ ≈ 1.5), since there is a large nucleation barrier to cargo coalescence.

This dependence on initial conditions demonstrates that kinetics quantitatively affects the size and morphology of assembled shells. However qualitative effects are limited by the degree of mismatch between the globule size and the shell preferred curvature; a large mismatch leads to budding of shells containing only part of the globule (S4 Fig).

To further evaluate whether assembly depends on kinetics or thermodynamics, we compared the dynamical simulation results against predictions of an equilibrium theory, based on rough estimates of equilibrium binding affinities and shell bending modulus values corresponding to our simulation parameters (section S2 Text). As shown in S6 and S7 Figs, the equilibrium dependence of the shell size on parameters exhibits similar qualitative trends as observed in the simulations, but the dynamical simulations exhibit larger variations in shell size than predicted at equilibrium.

#### Mechanisms of size selection

By comparing results from the equilibrium model and simulation results from two sets of initial conditions, we determine that the effect of cargo on shell size arises from the competition of several effects. The first two are equilibrium effects. Firstly, because only hexamers interact with the cargo, increasing the shell-cargo interaction increases the chemical potential of pentamers in the shell relative to hexamers. As noted above, decreasing pentamer adsorption favors larger shells, since there are only 12 pentamers in a complete shell (Fig 3 and S6 Fig at low *ε*_SC_). Similarly, decreasing the pentamer concentration *ρ*_p_ reduces pentamer insertion and thus decreases shell size (Fig 4 and S7 Fig). Secondly, however, increasing the shell-cargo interaction strength leads to a lower shell surface energy, which favors a larger surface-to-volume ratio and hence smaller shells. Above threshold values of *ε*_HH_ and *ε*_SC_, the second effect dominates (Fig 3 and S6 Fig at high *ε*_SC_). Due to these two competing effects, the equilibrium theory predicts a nonmonotonic dependence of the equilibrium shell size on *ε*_SC_. The equilibrium theory identifies other factors which affect the ratio of surface to bulk energy and thus shell size. For example, increasing the stoichiometric ratio of cargo to shell subunits decreases the cargo chemical potential thus favoring larger shells, consistent with a previous theoretical study on virus capsid assembly [57].

The tendency of the cargo to form spherical droplets also leads to kinetic effects on shell size, which depend on the relative rates of cargo coalescence and shell assembly. The sizes of the initial cargo globule and the final shell are correlated because the globule surface tension imposes a barrier to formation of shells with curvature radii that are smaller than the globule radius. Furthermore, since shell completion arrests globule coalescence, and stronger interactions drive faster assembly, the final size of the globule and the shell decrease with increasing *ε*_SC_ and *ε*_HH_. The assembly of larger shells in simulations started with the pre-equilibrated globule initial condition shows that this is at least partly a kinetic effect.

Finally, recall that above threshold values of *ε*_CC_ and *ε*_SC_, interactions are sufficiently strong that nucleation occurs throughout the system. Once complete (small) shells assemble around these nascent droplets, subsequent coarsening of globule-shell complexes is arrested on relevant timescales, resulting in a broad, non-equilibrium distribution of shell sizes (Fig 3B). The effect of each parameter on shell size is shown in Table 1.

**Table 1:** Effect of parameters on shell size.

Increasing parameter **decreases** shell size	
shell-cargo interaction*	*ε*_SC_
shell-shell interaction	*ε*_SS_
pentamer-hexamer affinity/hexamer-hexamer affinity	*ε*_ph_/*ε*_hh_
pentamer/hexamer stoichiametric ratio	*ρ*_p_/*ρ*_h_
shell subunit/cargo stoichiometric ratio	*ρ*_h_/*ρ*_c_
shell bending modulus (with spontaneous curvature)**	*κ*_s_
Increasing parameter **increases** shell size	
cargo-cargo interaction***	*ε*_CC_
shell bending modulus (with no spontaneous curvature)**	*κ*_s_

*At high *ε*_SC_, over-nucleation leads to a decrease in shell size.

**Increasing *κ*_s_ disfavors deviations from the shell spontaneous curvature, and thus favors small shells in the case of high spontaneous curvature or large shells in the case of low spontaneous curvature.

***Two step assembly leads to larger shells than single step pathways; however, sufficiently high values of *ε*_CC_ induce over-nucleation which decreases shell size.

### Shell subunits with no spontaneous curvature

**Fig 5:**
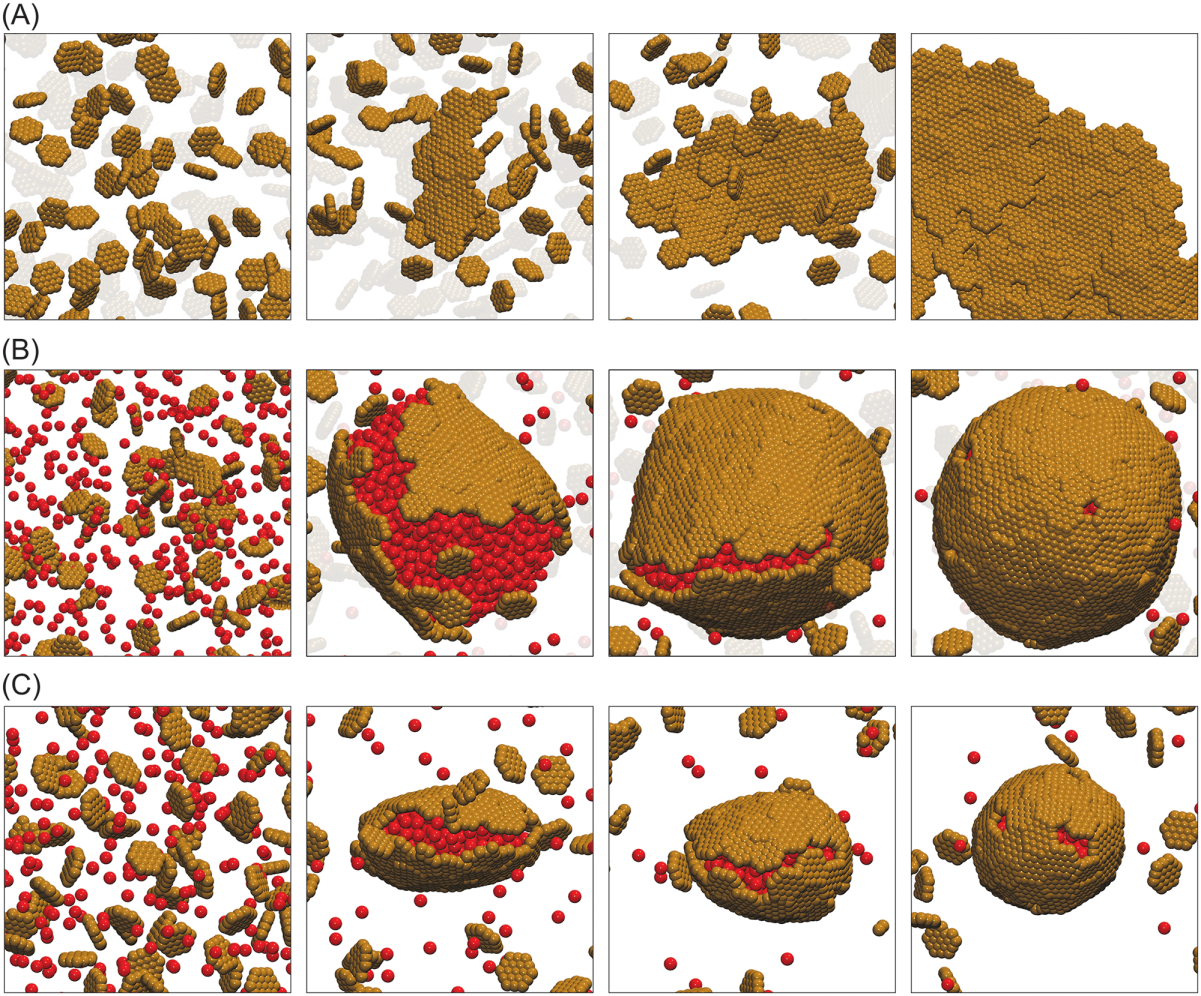
Snapshots of assembly trajectories for hexamer subunits with zero spontaneous curvature. **(A)** Assembly with no cargo, for *ε*_HH_ = 2.5, and shell bending modulus parameter *ε*_angle_ = 0.1 (shell bending modulus *κ*_s_ ≈ 20*k*_B_*T*). **(B)** Assembly with cargo, for *ε*_HH_ = 1.8, *ε*_SC_ = 7.0, and *ε*_angle_ = 0.08 (*κ*_s_ ≈ 18*k*_B_*T*). The final shell has 231 and 2261 hexamers and cargo particles respectively, as well as 12 pentameric vacancies. **(C)** Assembly with cargo in a small system with low shell bending modulus, for *ε*_HH_ = 1.8, *ε*_SC_ = 7.0, and *ε*_angle_ = 0.015 (*κ*_s_ ≈ 3*k*_B_*T*). The final shell has 71 and 361 hexamers and cargo particles respectively, 8 pentameric vacancies, and 2 double vacancies. An example of a double vacancy is visible in the front of the final frame. Animations of the trajectories in (B) and (C) are shown in S4 and S5 videos respectively.

We now consider the opposite limit: a system of ‘flat’ hexamer subunits, which have zero spontaneous curvature and thus favor formation of flat sheets (Fig 5A). Fig 5B shows a typical assembly trajectory for flat subunits with *ε*_CC_ = 1.7, in which the cargo rapidly coalesces followed by adsorption and assembly of the hexamers. Interestingly, the shapes of assembly intermediates reflect the lack hexamer spontaneous curvature—hexamers initially assemble into flat sheet wrapped around the globule, deforming the spherical globule into a cigar shape. Eventually the two sides of the sheet meet, creating a seam with an unfavorable line tension due to unsatisfied subunit contacts. As the seam gradually fills in, the elastic energy associated with such an acute deformation forces the complex toward a more spherical shape. As in systems with spontaneous curvature, the hexamer shells exhibit the 12 five-fold vacancy defects required by topology. If pentamers are present they eventually fill these holes (as in Fig 2 above), but for simplicity we consider systems containing only hexamers here. The large shells are roughly but not perfectly icosahedral, presumably reflecting slow defect reorganization on assembly timescales.

The size of the assembled shell is limited by the finite system size of our simulations. Importantly, the same limitation occurs within cells when the cargo undergoes phase separation into a single complex whose size is limited by the enzyme copy number (*e.g*. the procarboxysome precursor to carboxysome assembly [39, 40]). We therefore investigated the dependence of assembly morphologies on system size, as a function of the shell bending modulus, *κ*_s_ (controlled by the parameter *ε*_angle_). Specifically, at each value of *κ*_s_ we performed a series of simulations in which the maximum size of the cargo globule was controlled by changing the system size with fixed total cargo concentration and hexamer chemical potential (section Materials and methods). An example assembly trajectory for a small system is shown in Fig 5C.

**Fig 6:**
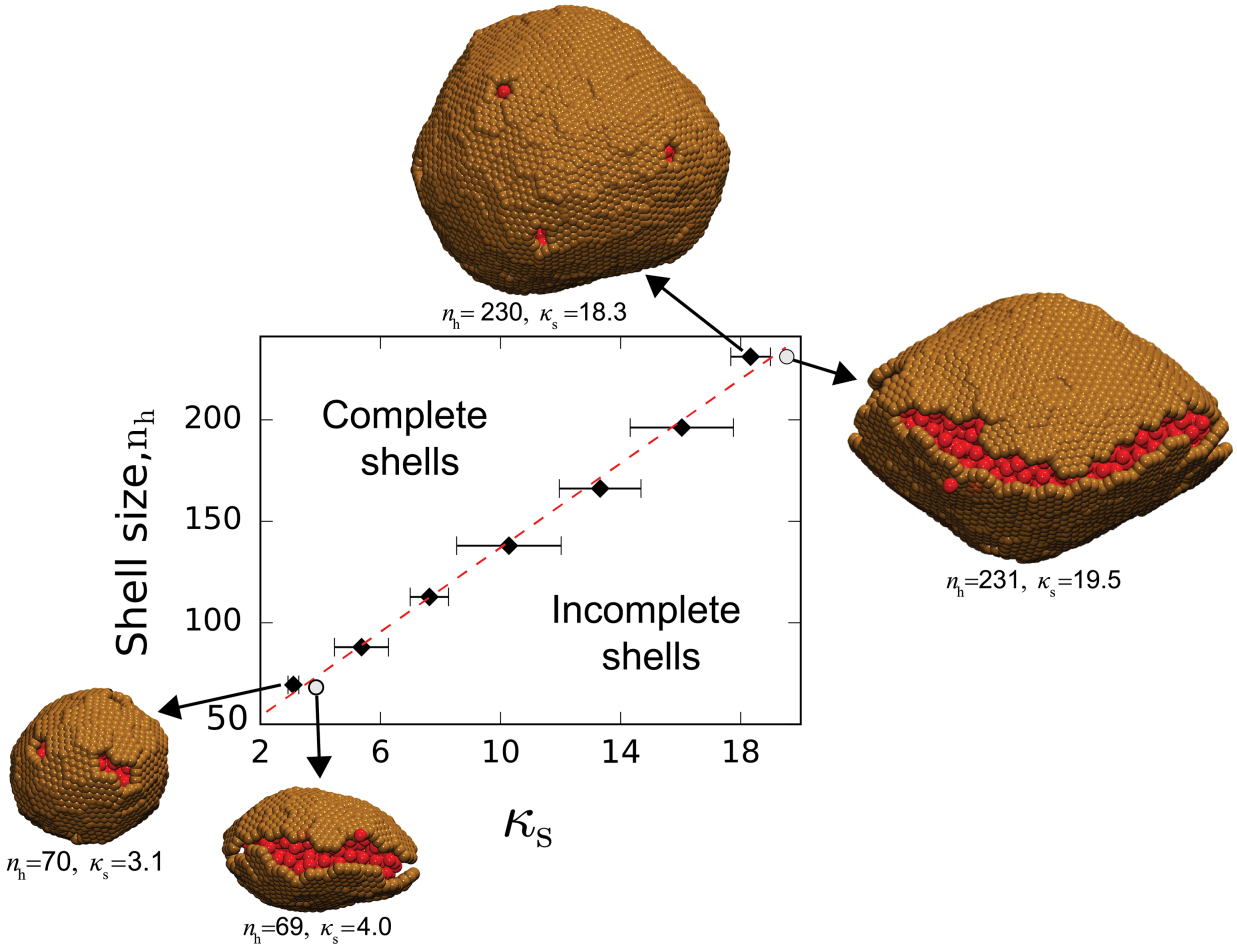
Size and morphology of shells assembled from subunits with no spontaneous curvature, for varying system sizes and shell bending modulus *κ*_s_. The y-axis gives the number of subunits in the largest cluster at the final simulation frame. Black diamonds correspond to Brownian dynamics simulation results for the smallest system size in which a complete shell formed, and the dashed line shows the best fit of Eq (1) to this data. The snapshots show examples of the final morphology at indicated parameter values. Two snapshots are shown of shells just below the threshold size for completion, with corresponding parameters indicated by circles. Other parameters are *ε*_CC_ = 1.7, *ε*_HH_ = 1.8, and *ε*_SC_ = 7.0.

As shown in Fig 6, we observe a minimum globule size required for complete shell assembly, which linearly increases with *κ*_s_. We observe complete wrapping for all system sizes above this threshold. Below the threshold size, assembly stalls with one or more open seams remaining; examples of this configuration are shown for a low and high bending modulus in Fig 6. Interestingly, while the pentameric defects are roughly equally spaced within large shells, small shells assembled with extremely low values of *κ*_s_ tend to exhibit adjacent vacancy pairs (Fig 5C, final frame). This defect morphology focuses curvature in a region with no elastic energy (the vacancy) while reducing the number of unsatisfied hexamer edges.

To understand these results, in section S2 Text we present a calculation of the equilibrium shell size distribution for subunits with no spontaneous curvature and stoichiometrically limiting cargo. We restrict the ensemble to spherical shells as observed in the simulations. While the aggregates are large and polydisperse without cargo, the calculation shows that cargo leads to a minimum free energy spherical shell size (S8 and S9 Figs).

The linear relationship between minimum shell size and bending modulus can be understood from our equilibrium model by comparing the excess free energy difference ΔΩ_wrap_ between the complete shell and an unwrapped globule (see section S2 Text). For the simulated conditions, the size and shape of the cargo globule is essentially the same in each of these states, and thus the free energy difference for a globule wrapped by *n*_h_ hexamers in Eq. (S2.9) simplifies to

$$\begin{array}{c} \Delta \Omega_{\text{wrap}}=8\pi \kappa_{\text{s}}+\Delta G_{\text{p}}+\Delta \mu_{\text{h}}n_{\text{h}} \end{array}$$ (1)

with Δ*μ*_h_ = *g*_hh_ + *g*_hc_ − *μ*_h_, Δ*G*_p_ as the free energy due to the 12 pentameric vacancies, *g*_hh_(*ε*_HH_) as the hexamer-hexamer interaction free energy, *g*_hc_(*ε*_SC_) as the hexamer-cargo free energy, and *μ*_h_ = *k*_B_*T* log(*ρ*_h_) the chemical potential of unassembled hexamers at concentration *ρ*_h_. The term 8*πκ*_s_ describes the bending energy of the complete shell. The minimum globule size n* corresponds to the locus of parameter values at which ΔΩ_wrap_ = 0, giving

$$\begin{array}{c} n^{*}=\frac{8\pi }{-\Delta \mu_{\text{h}}}\kappa_{\text{s}}+\frac{\Delta G_{\text{p}}}{-\Delta \mu_{\text{h}}} \end{array}$$ (2)

A linear fit to the simulation results for n* results in Δ*μ*_h_ = −2.4 and Δ*G*_p_ = 80.5*k*_B_*T*, or 6.7*k*_B_*T* per pentameric defect. Plugging in *ρ*_h_ = 10^−3^ subunits/$\sigma_{0}^{3}$ and *g*_hc_ = −8.1*k*_B_*T* for *ε*_SC_ = 7.0 (using the estimate from Perlmutter *et al*. [42]) then results in *g*_hh_ ≈ −0.45*k*_B_*T*. This value and the fit value of Δ*G*_p_ are reasonably close to interactions estimated from the relationship between the shell-shell dimerization free energy *g*_hh_ and potential well-depth *ε*_HH_ for a similar model in Perlmutter *et al*. [42]. Thus, the simulation results are consistent with the minimum stable shell size predicted by the theory.

### Conclusions

We have used computational and theoretical modeling to investigate factors that control the assembly of a protein shell around a fluid cargo. We have focused on two limiting regimes of protein interaction geometries—high spontaneous curvature that drives the formation of small shells, and zero spontaneous curvature that favors assembly of flat sheets or polydisperse shells. In both regimes the presence of cargo can significantly alter the size distribution of assembled shells. For high spontaneous curvature, encapsulated cargo tends to increase shell size, whereas for shell proteins with low (or zero) spontaneous curvature cargo templating provides a mechanism to drive shell curvature and thus tends to reduce shell size. These results could provide a qualitative explanation for experimental observations on different systems in which full microcompartment shells were either larger or smaller than empty shells [23, 28, 31, 37, 38].

Our simulations identify a combination of kinetic and thermodynamic mechanisms governing microcompartment size control. At equilibrium, the shell size is determined by the stoichiometry between cargo and shell subunits, with an excess of cargo or shell protein respectively favoring larger or smaller shells. Similarly, a high surface energy (high cargo surface tension and weak shell-cargo interactions) favors larger shells whereas a strong shell bending modulus favors shells closer to the preferred size. Although dynamical simulations exhibit similar qualitative trends to these equilibrium results, we observe significant kinetic effects as well. Fast cargo coalescence relative to rates of shell assembly favors larger shells, since closure of an assembling shell prevents further cargo aggregation. Thus, the shell size is strongly correlated to the assembly pathway, with two-step assembly leading to larger shells than single-step pathways. Although many factors likely control shell size in biological systems, this result is consistent with the observations of small empty shell assemblies [23, 28, 31, 37] and the fact that *β*-carboxysomes (which assemble by two step pathways [39, 40]) tend to be larger and more polydisperse than *α*-carboxysomes (which experiments suggest assemble by one-step pathways [33, 41]).

Our results for shell proteins without spontaneous curvature build upon Rotskoff and Geissler [58], which identified a kinetic mechanism in which cargo templating drives shell curvature, and shell closure eventually arrests assembly. Their mechanism proceeds by two-step assembly, with initial nucleation of a cargo globule followed by assembly of shell subunits, but requires that rates of subunit arrival are at least 10 times faster than cargo arrival rates [58]. However, it is unclear how many physical microcompartment systems may fit this criteria, and our results suggest other mechanisms may play important roles in microcompartment assembly. Firstly, if cargo is stoichiometrically limiting then the finite-pool mechanism can result in finite shell sizes, with the coalesced cargo still providing a template for shell curvature. Secondly, subunits with spontaneous curvature can form complete shells even under conditions of excess cargo or fast coalescence rates that lead to large cargo aggregates (Fig 3D), as observed for carboxysome assembly in cells [39]. Thus, biological microcompartments with some degree of preferred shell curvature could robustly assemble over a much wider parameter space than systems without spontaneous curvature. Intriguingly, the recent atomic-resolution microcompartment structure from Sutter *et al*. [31] suggests that different hexamer or pseudo-hexamer species have different preferred subunit-subunit angles, and thus the spontaneous curvature may depend on the shell composition. We will investigate this in a future work.

The importance of spontaneous curvature to a particular BMC system could be investigated by comparing our computational predictions to experimental shell size distributions measured for varying cargo/shell protein stoichiometries and interaction strengths. While such tests would be most straightforward to perform in vitro, they could be performed in vivo by varying expression levels of various shell proteins or the enzymatic cargoes. Of particular interest would be a comparison between the shell size distribution in the presence and absence of cargo. However, note that we have focused on extreme limits (high spontaneous curvature or zero spontaneous curvature); systems with moderate shell spontaneous curvature may exhibit less dramatic cargo effects. Also note that the effective shell spontaneous curvature depends on the stoichiometries of different shell protein species; *e.g*., overexpressing pentamers would shift the size distribution toward smaller shells (Fig 2D).

These results have implications for targeting new core enzymes to BMC interiors. Recent experiments have shown that alternative cargoes can be targeted to BMC interiors by incorporating encapsulation peptides that mediate cargo-shell interactions, but that relatively small amounts of cargo were packaged [21–23, 96]. Our previous simulations showed that assembly of full shells requires both cargo-shell and cargo-cargo (direct or mediated) interactions. Here, we see that the strength of cargo-cargo interactions can not only affect the efficiency of cargo loading, but also the size of the containing shell.

## Supporting information

S1 Fig Fraction of Brownian dynamics trials at each parameter set that lead to at least one complete shell. A complete shell is defined as a structure in which all pentamers and hexamers have respectively five and six interactions with neighbors. Results are shown as a function of *ε*_SC_ at indicated values of *ε*_CC_. Other parameters are *ε*_HH_ = 1.8, *ε*_PH_/*ε*_HH_ = 1.3, *ρ*_p_/*ρ*_h_ = 0.5, and *κ*_s_ = 10*k*_B_*T*.
(TIF)

S2 Fig Quality of shells. Ratio of complete shells to the total number of shells with at least 32 subunits as a function of *ε*_SC_ at indicated values of *ε*_CC_. Other parameters are as in S1 Fig.
(TIF)

S3 FigThe degree of icosahedral symmetry increases with shell size for full shells.The bond order parameter *Q*_6_ of Ref. [97] is shown as a function of the number of subunits in a shell, with $Q_{l}={\left[\frac{4\pi }{2l+1}\sum_{m=-l}^{l}{\left|{\bar{Q}}_{lm}\right|}^{2}\right]}^{1/2},{\bar{Q}}_{lm}\equiv \langle Q_{lm}\left(r\right)\rangle$ where the average is taken over all the geometric center of each pentamer **r**, and *Q*_*lm*_(**r**) is the (*lm*th) spherical harmonic of **r**. Results are normalized by the value for perfect icosahedral symmetry, *Q*_6_ = 0.663, and blue circles correspond to the complete shells from the simulations used for Fig 3, while black triangles correspond to empty shells.(TIF)

S4 Fig Snapshots of assembly around a pre-equilibrated cargo globule. These snapshots are from Brownian dynamics simulations that used an alternative initial condition (described in the text), in which cargo particles were allowed to equilibrate before introduction of shell subunits. **(A)** With *ε*_SC_ = 8.0, *ε*_HH_ = 2.0, *ρ*_p_/*ρ*_h_ = 0.6, *ε*_PH_/*ε*_HH_ = 1.5, and *κ*_s_ = 16*k*_B_*T*, small shells assemble and bud from the globule. At this moderate shell-cargo affinity, pentamers rapidly associate with adsorbed hexamers, driving high shell curvature. The final shells have 44-63 subunits, encapsulating 133-274 cargo particles. **(B)** With stronger shell-cargo interactions (*ε*_SC_ = 10, other parameters as in (A)), hexamers adsorb rapidly and exclude pentamers from the globule. Eventually there are 12 vacancies in the hexamer lattice that are filled by pentamers. The final shell has 104 subunits encapsulating 641 cargo particles. **(C)** Further increasing the shell cargo interaction (*ε*_SC_ = 12, other parameters as in (A)) leads to multiple nucleation events and polydisperse shell. The simulation results in four complete shells containing 37-92 subunits and 116-532 cargo particles.
(TIF)

S5 Fig Comparison of the mean shell size for BD simulations started from the homogeneous initial condition and pre-equilibrated globule initial conditions for varying *ε*_SC_. Other parameters are *ε*_CC_ = 1.5, *ε*_HH_ = 2.0, *ε*_PH_/*ε*_HH_ = 1.5, *ρ*_p_/*ρ*_h_ = 0.5, and *ε*_angle_ = 1.0 (*κ*_s_ ≈ 16*k*_B_*T*).
(TIF)

S6 Fig Predictions from the equilibrium model (Eqs. (S2.1)– (S2.3) and (S2.8)) for the mean shell size as a function of the cargo-cargo and shell-cargo affinities. **(A)** Results are shown for parameters at which at least 1% of subunits are in shells, for *ε*_HH_ = 1.8, and shell bending modulus *κ*_s_ = 10*k*_B_*T*. Cargo and shell volume fractions are the same as in Fig 3. **(B)** Mean and standard deviation of the equilibrium shell size distribution as a function of cargo-cargo affinity, maximized over shell-cargo affinity. Other parameters are as in (A).
(TIF)

S7 Fig Mean shell size predicted by the equilibrium theory (Eqs. (S2.1)– (S2.3) and (S2.8)) as a function of pentamer/hexamer stoichiometry ratio *ρ*_p_/*ρ*_h_ and pentamer/hexamer affinity ratio *ε*_PH_/*ε*_HH_. The theory parameters are calculated to approximately match the simulation parameters in Fig 4 (see section S2 Text), with *ε*_HH_ = 1.8, *κ*_s_ = 10, *ε*_CC_ = 1.65, and *ε*_SC_ = 10.0.
(TIF)

S8 Fig Equilibrium theory prediction of mean shell size for subunits with no spontaneous curvature restricted to icosahedral shells, in the presence (red squares) and absence (blue triangles) of cargo. The mean shell size is shown as a function of hexamer concentration, calculated from Eqs. (S2.8) and (S2.12) with hexamer-cargo affinity *g*_hc_ = −8.1 (corresponding to *ε*_SC_ = 7.0, see Ref. [42]), and *κ*_s_ = 20*k*_B_*T*. The hexamer-hexamer affinity *g*_hh_ = −0.45 and the energy of 12 pentameric vacancies Δ*G*_p_ = 80.5*k*_B_*T* were obtained from the fit to the simulations in Fig 6.
(TIF)

S9 Fig Equilibrium shell size distribution for subunits with no spontaneous curvature. (A) Empty shells and (B) With cargo, under conditions of excess shell subunits (limiting cargo). Size distributions are obtained by solving Eq. (S2.12), with Δ*μ*_c_ = 0.18, Δ*G*_p_ = 80, and *κ*_s_ = 20*k*_B_*T*. Other parameters are from the calculations in Ref. [42] for *ε*_SC_ = 7.0, *ε*_CC_ = 1.7, and *ε*_HH_ = 1.8.
(TIF)

S10 Fig Total interaction energy of a complete shell with preferred *T* = 3 curvature, measured in BD simulations with different values of *ε*_angle_. The shell has 98 hexamers and 12 pentamers, and other parameters are *ε*_HH_ = 1.8, *ε*_SC_ = 9.0, and *ε*_CC_ = 1.5.
(TIF)

S1 Video Assembly of empty *T* = 3 shells from shell subunits with spontaneous curvature. Parameters are hexamer-hexamer interaction strength *ε*_HH_ = 2.6, ratio of pentamer/hexamer affinity *ε*_PH_/*ε*_HH_ = 1.3, and pentamer/hexamer stoichiometric ratio *ρ*_p_/*ρ*_h_ = 0.5.
(MP4)

S2 Video One-step assembly with moderate cargo-cargo interaction strength, *ε*_CC_ = 1.5. The final structure has 68 hexamers, 12 pentamers, and 408 encapsulated cargo particles. Other parameters are hexamer-hexamer interaction strength *ε*_HH_ = 1.8, ratio of pentamer/hexamer affinity *ε*_PH_/*ε*_HH_ = 1.3, *ρ*_p_/*ρ*_h_ = 0.5, and shell-cargo affinity *ε*_SC_ = 8.75. Please be aware that this file is over 20MB.
(MP4)

S3 Video Two-step assembly pathway for strong cargo-cargo affinity *ε*_CC_ = 1.65. The final structure has 167 hexameters, 12 pentamers, and 1520 encapsulated cargo particles. Other parameters are *ε*_HH_ = 1.8, *ε*_SC_ = 8.5, *ε*_PH_/*ε*_HH_ = 1.3, and *ρ*_p_/*ρ*_h_ = 0.5. Please be aware that this file is over 20MB.
(MP4)

S4 Video Assembly of subunits with no spontaneous curvature around cargo, for *ε*_HH_ = 1.8, *ε*_SC_ = 7.0, and *ε*_angle_ = 0.08. The final shell has 231 and 2261 hexamers and cargo particles respectively, as well as 12 pentameric vacancies. Please be aware that this file is over 20MB.
(MP4)

S5 Video Assembly of subunits with no spontaneous curvature around cargo in a small system with low shell bending modulus, for *ε*_HH_ = 1.8, *ε*_SC_ = 7.0, and *ε*_angle_ = 0.015. The final shell has 71 and 361 hexamers and cargo particles respectively, 8 pentameric vacancies, and 2 double vacancies. An example of a double vacancy is visible in the front of the final frame. Please be aware that this file is over 20MB.
(MP4)

S1 Text Model details. (PDF)

S2 Text Thermodynamics. (PDF)
