# Correction: The role of the encapsulated cargo in microcompartment assembly

**DOI:** 10.1371/journal.pcbi.1006901

**Published:** 2019-05-02

**Authors:** The PLOS Computational Biology Staff

In Fig 3 the “overnucleated” was duplicated, and shown instead of the “incomplete” snapshot. The correct figure is below:

**Fig 3:**
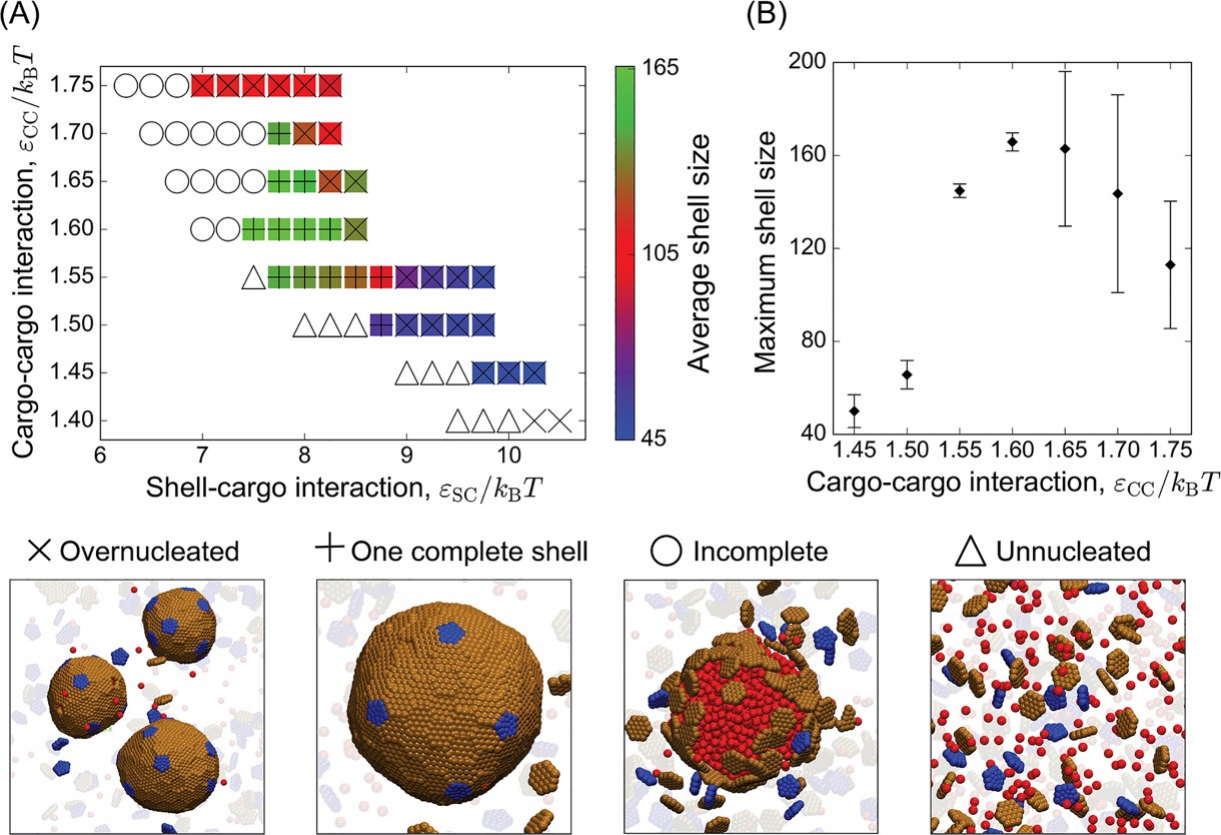
Dependence of the mean shell size and most probable morphology on the cargo-cargo and subunit-cargo affinities (*ε*_CC_ & *ε*_SC_). **(A)** The mean shell size (number of hexamers + 12 pentamers) is indicated by the color bar, and the predominant morphology is indicated by symbols, with a snapshot corresponding to each morphology shown on the bottom. **(B)** The mean shell size maximized over *ε*_SC_ is shown as a function of *ε*_CC_. Other parameters in (A) and (B) are *ε*_HH_ = 1.8, *ρ*_p_*/ρ*_h_ = 0.5, *ε*_PH_*/ε*_HH_ = 1.3, and *κ*_s_ = 10*k*_B_*T*.
