# Supplementary figures and images for: The role of the encapsulated cargo in microcompartment assembly
Source: PLoS Comput Biol. 2018 Jul 31;14(7):e1006351. doi: 10.1371/journal.pcbi.1006351 (PMC6086489; doi:10.1371/journal.pcbi.1006351)

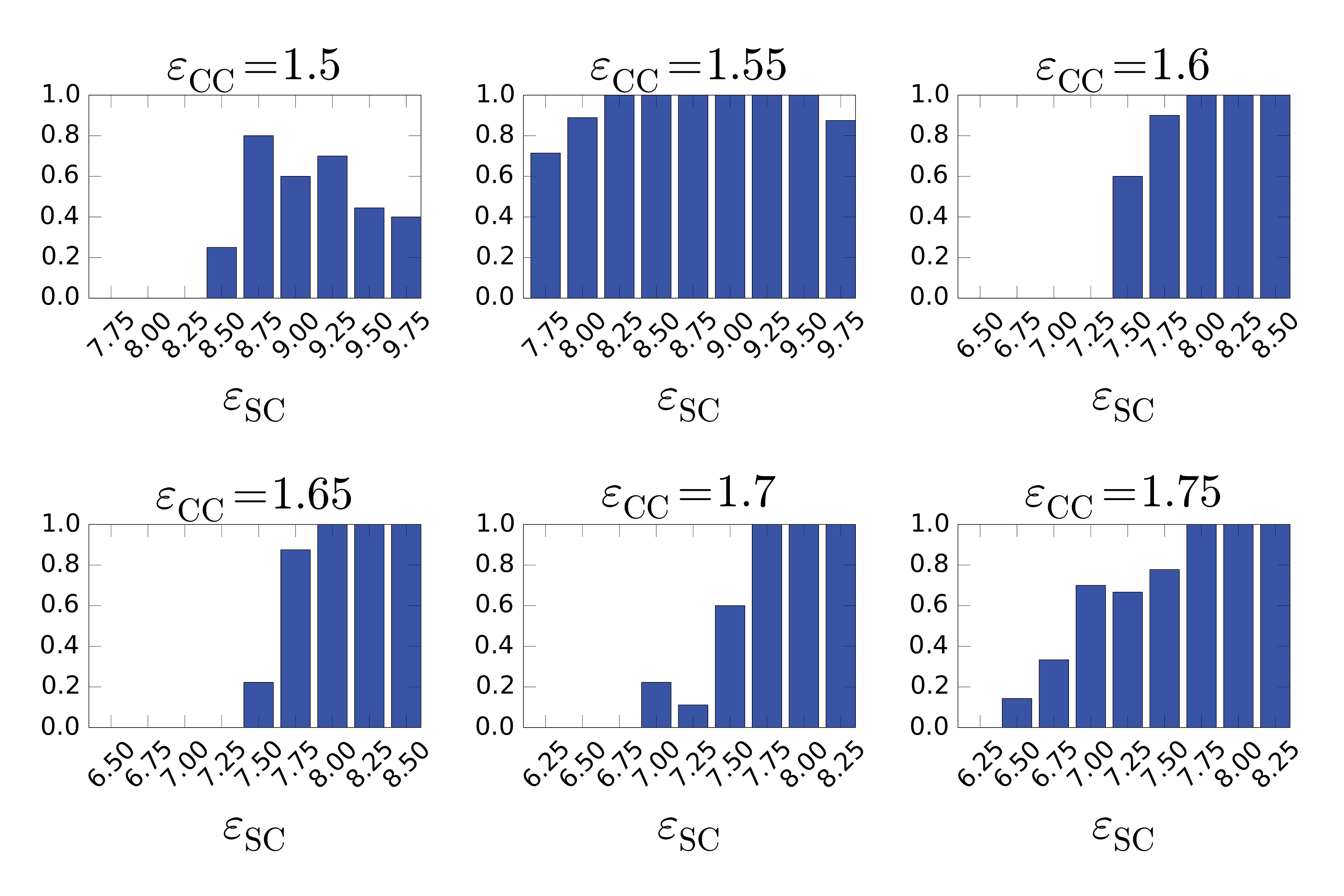

Supplement: S1 Fig — A complete shell is defined as a structure in which all pentamers and hexamers have respectively five and six interactions with neighbors. Results are shown as a function of εSC at indicated values of εCC. Other parameters are εHH = 1.8, εPH/εHH = 1.3, ρp/ρh = 0.5, and κs = 10kBT. (TIF) [file pcbi.1006351.s001.tif]

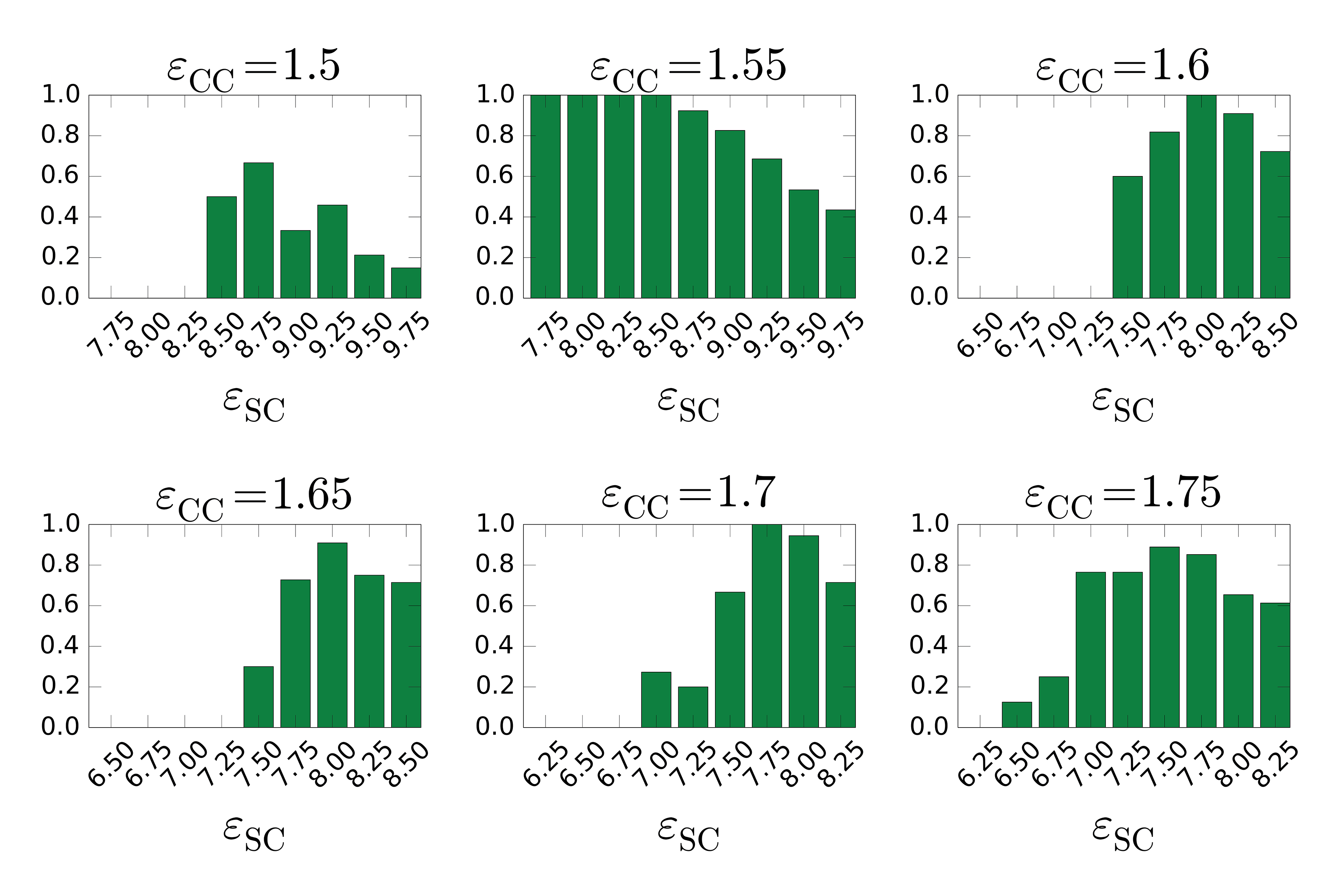

Supplement: S2 Fig — Ratio of complete shells to the total number of shells with at least 32 subunits as a function of εSC at indicated values of εCC. Other parameters are as in S1 Fig. (TIF) [file pcbi.1006351.s002.tif]

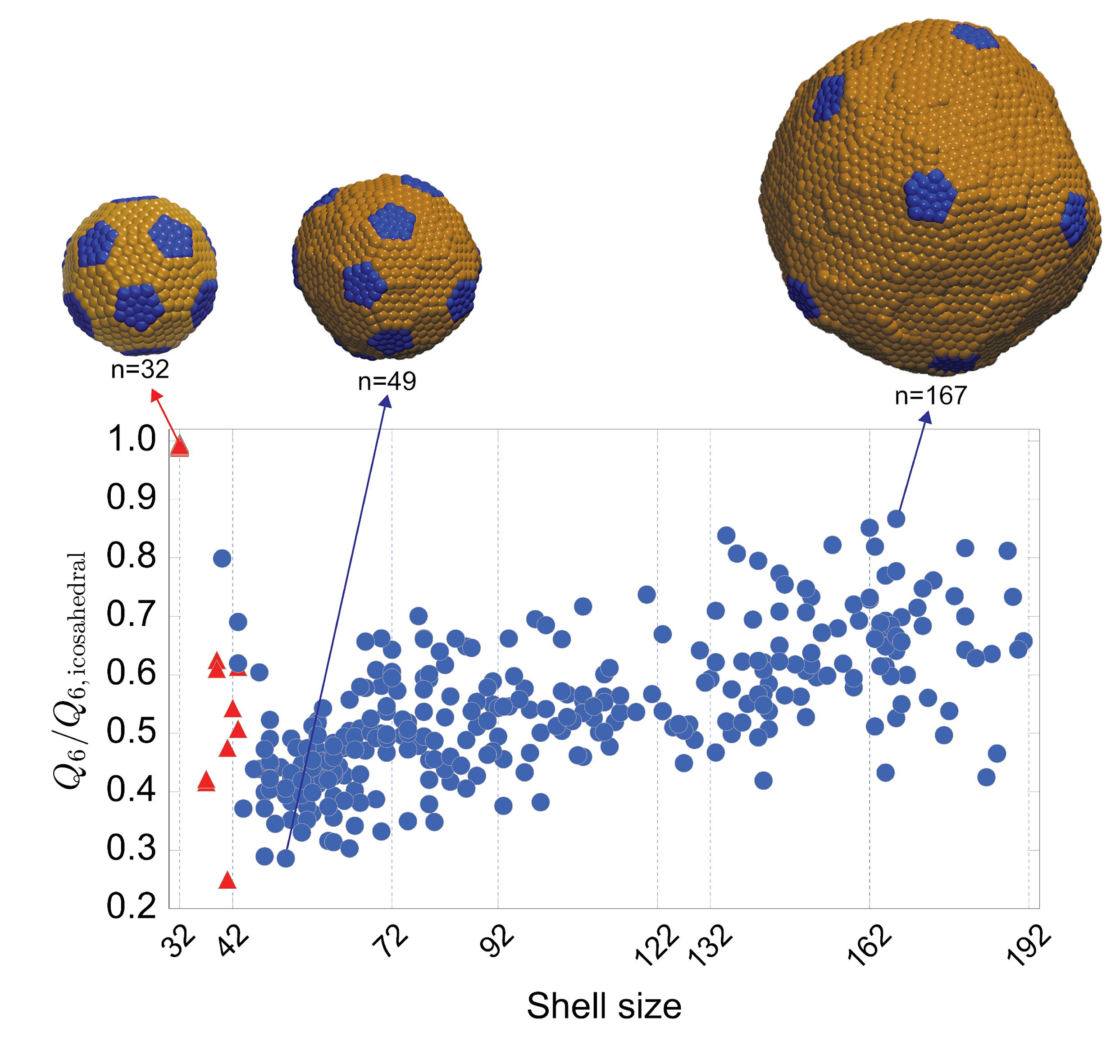

Supplement: S3 Fig — The bond order parameter Q6 of Ref. [97] is shown as a function of the number of subunits in a shell, with Ql=[4π2l+1∑m=−ll|Q¯lm|2]1/2,Q¯lm≡〈Qlm(r)〉 where the average is taken over all the geometric center of each pentamer r, and Qlm(r) is the (lmth) spherical harmonic of r. Results are normalized by the value for perfect icosahedral symmetry, Q6 = 0.663, and blue circles correspond to the complete shells from the simulations used for Fig 3, while black triangles correspond to empty shells. (TIF) [file pcbi.1006351.s003.tif]

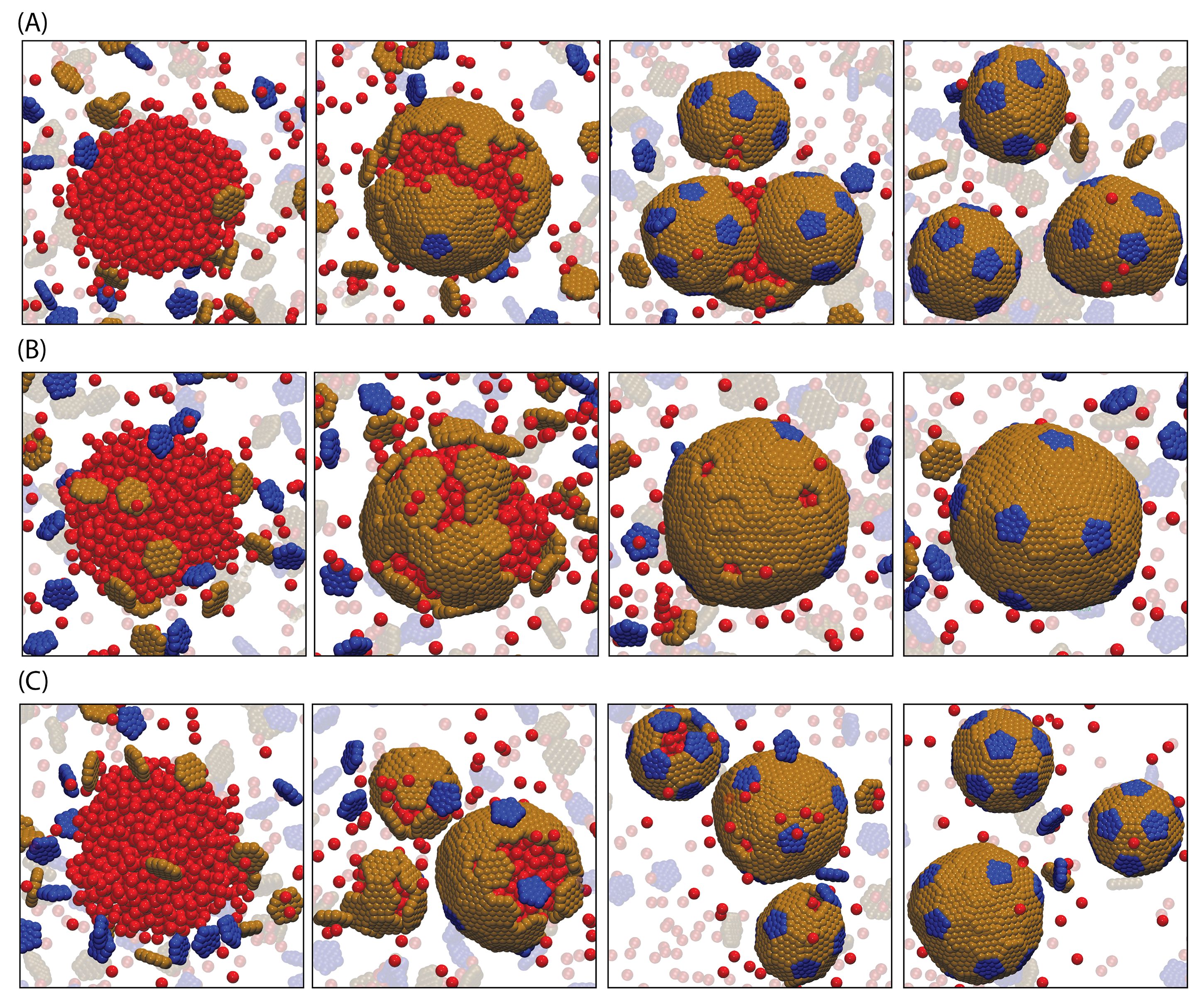

Supplement: S4 Fig — These snapshots are from Brownian dynamics simulations that used an alternative initial condition (described in the text), in which cargo particles were allowed to equilibrate before introduction of shell subunits. (A) With εSC = 8.0, εHH = 2.0, ρp/ρh = 0.6, εPH/εHH = 1.5, and κs = 16kBT, small shells assemble and bud from the globule. At this moderate shell-cargo affinity, pentamers rapidly associate with adsorbed hexamers, driving high shell curvature. The final shells have 44-63 subunits, encapsulating 133-274 cargo particles. (B) With stronger shell-cargo interactions (εSC = 10, other parameters as in (A)), hexamers adsorb rapidly and exclude pentamers from the globule. Eventually there are 12 vacancies in the hexamer lattice that are filled by pentamers. The final shell has 104 subunits encapsulating 641 cargo particles. (C) Further increasing the shell cargo interaction (εSC = 12, other parameters as in (A)) leads to multiple nucleation events and polydisperse shell. The simulation results in four complete shells containing 37-92 subunits and 116-532 cargo particles. (TIF) [file pcbi.1006351.s004.tif]

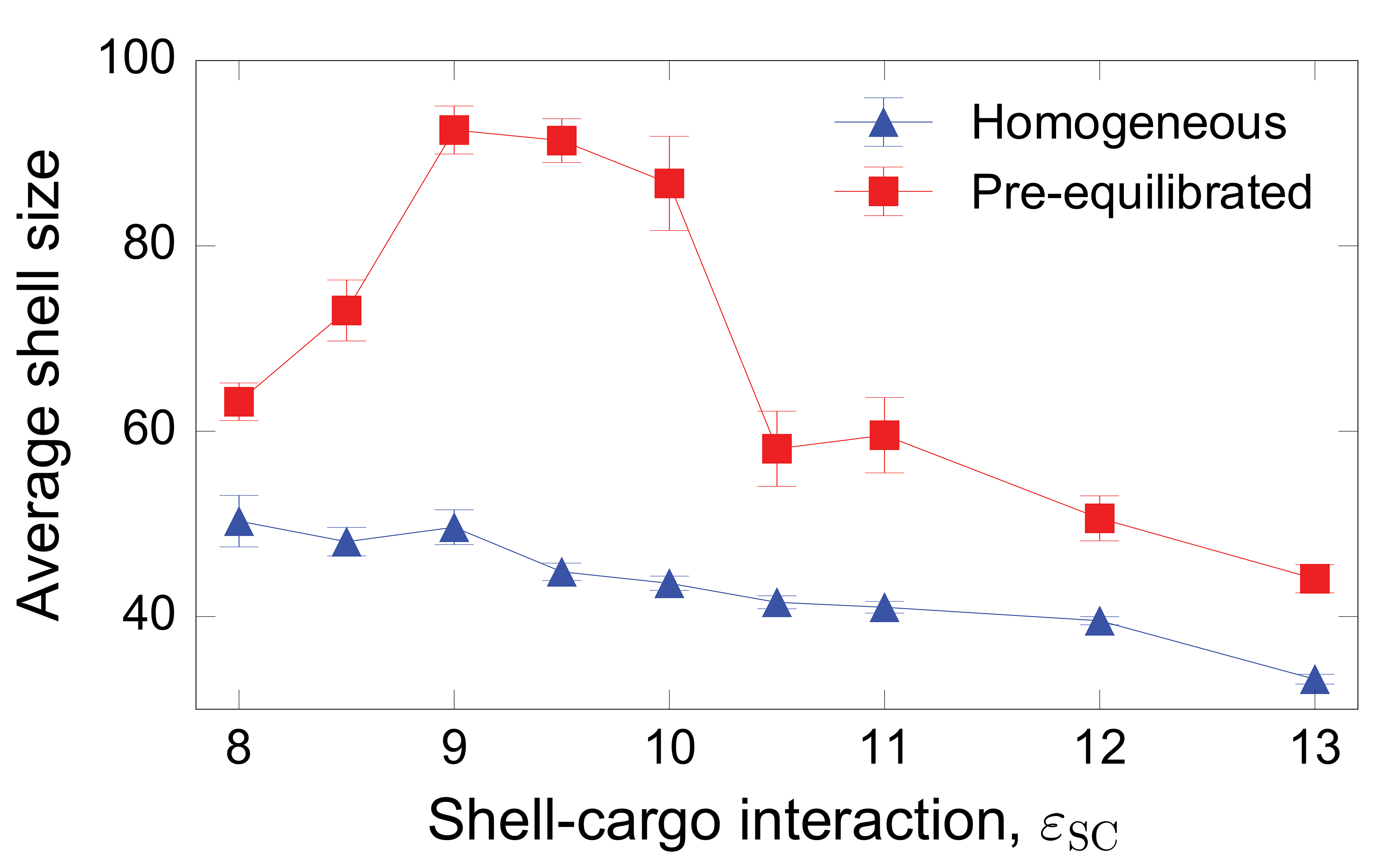

Supplement: S5 Fig — Other parameters are εCC = 1.5, εHH = 2.0, εPH/εHH = 1.5, ρp/ρh = 0.5, and εangle = 1.0 (κs ≈ 16kBT). (TIF) [file pcbi.1006351.s005.tif]

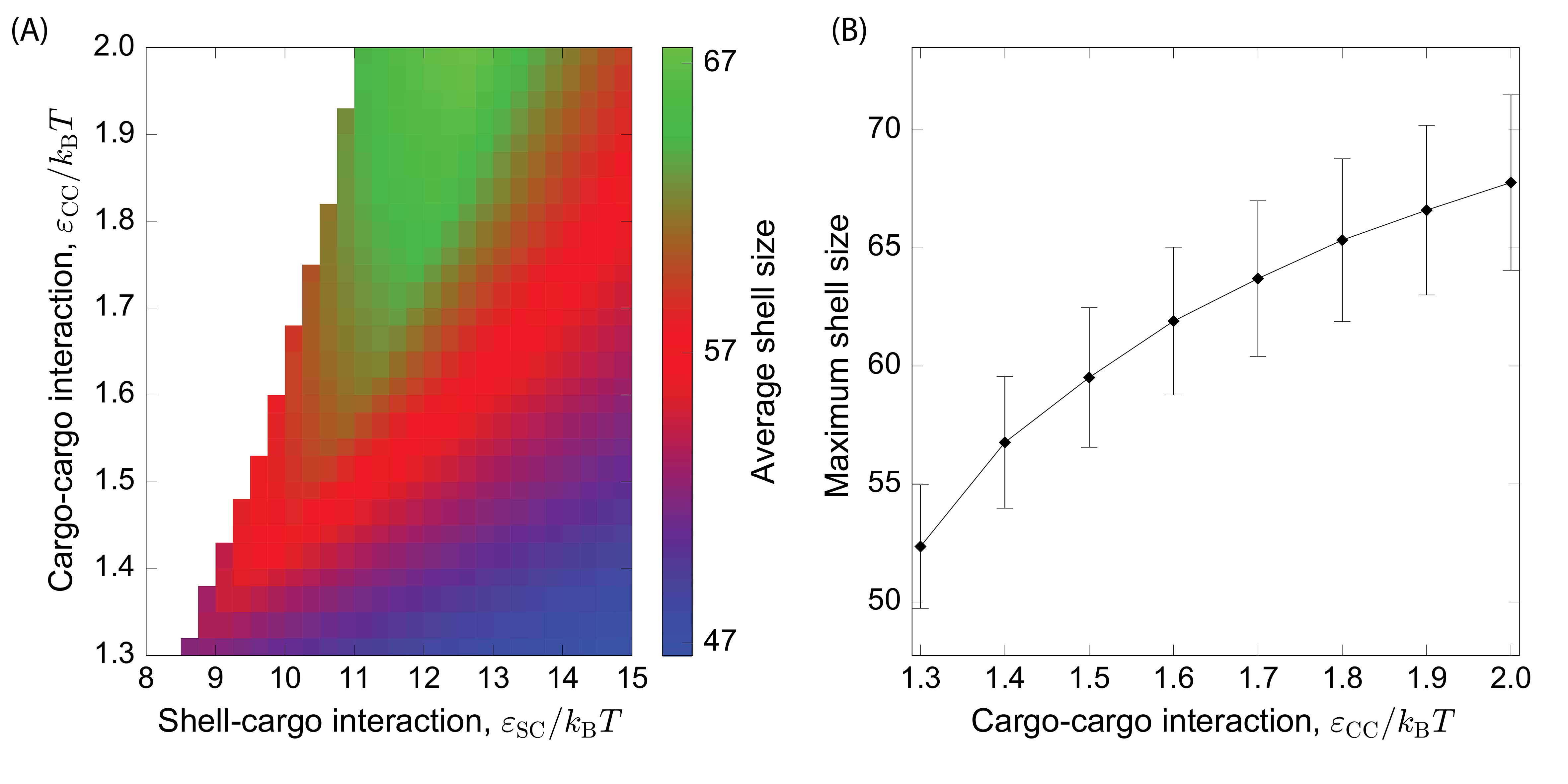

Supplement: S6 Fig — (A) Results are shown for parameters at which at least 1% of subunits are in shells, for εHH = 1.8, and shell bending modulus κs = 10kBT. Cargo and shell volume fractions are the same as in Fig 3. (B) Mean and standard deviation of the equilibrium shell size distribution as a function of cargo-cargo affinity, maximized over shell-cargo affinity. Other parameters are as in (A). (TIF) [file pcbi.1006351.s006.tif]

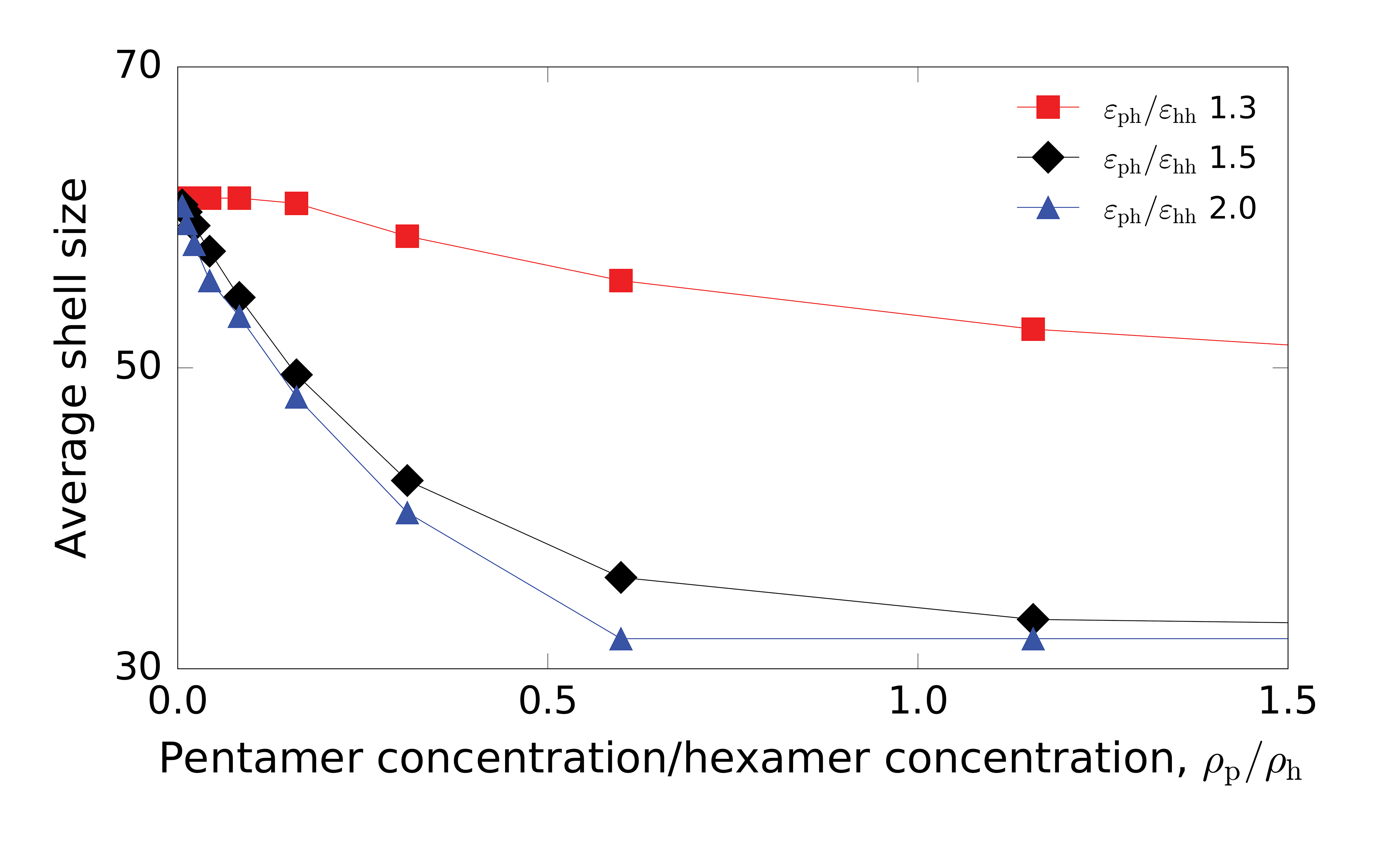

Supplement: S7 Fig — The theory parameters are calculated to approximately match the simulation parameters in Fig 4 (see section S2 Text), with εHH = 1.8, κs = 10, εCC = 1.65, and εSC = 10.0. (TIF) [file pcbi.1006351.s007.tif]

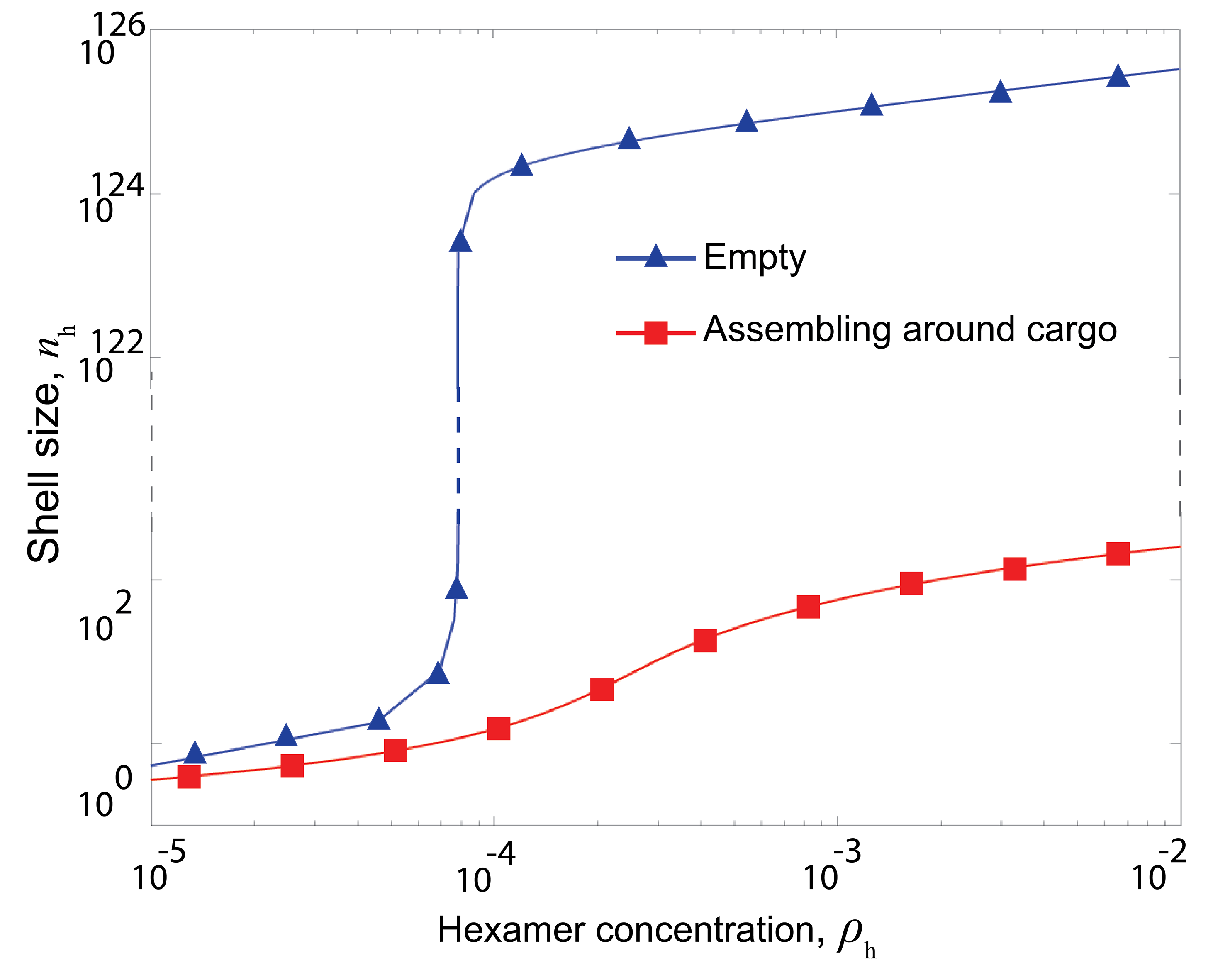

Supplement: S8 Fig — The mean shell size is shown as a function of hexamer concentration, calculated from Eqs. (S2.8) and (S2.12) with hexamer-cargo affinity ghc = −8.1 (corresponding to εSC = 7.0, see Ref. [42]), and κs = 20kBT. The hexamer-hexamer affinity ghh = −0.45 and the energy of 12 pentameric vacancies ΔGp = 80.5kBT were obtained from the fit to the simulations in Fig 6. (TIF) [file pcbi.1006351.s008.tif]

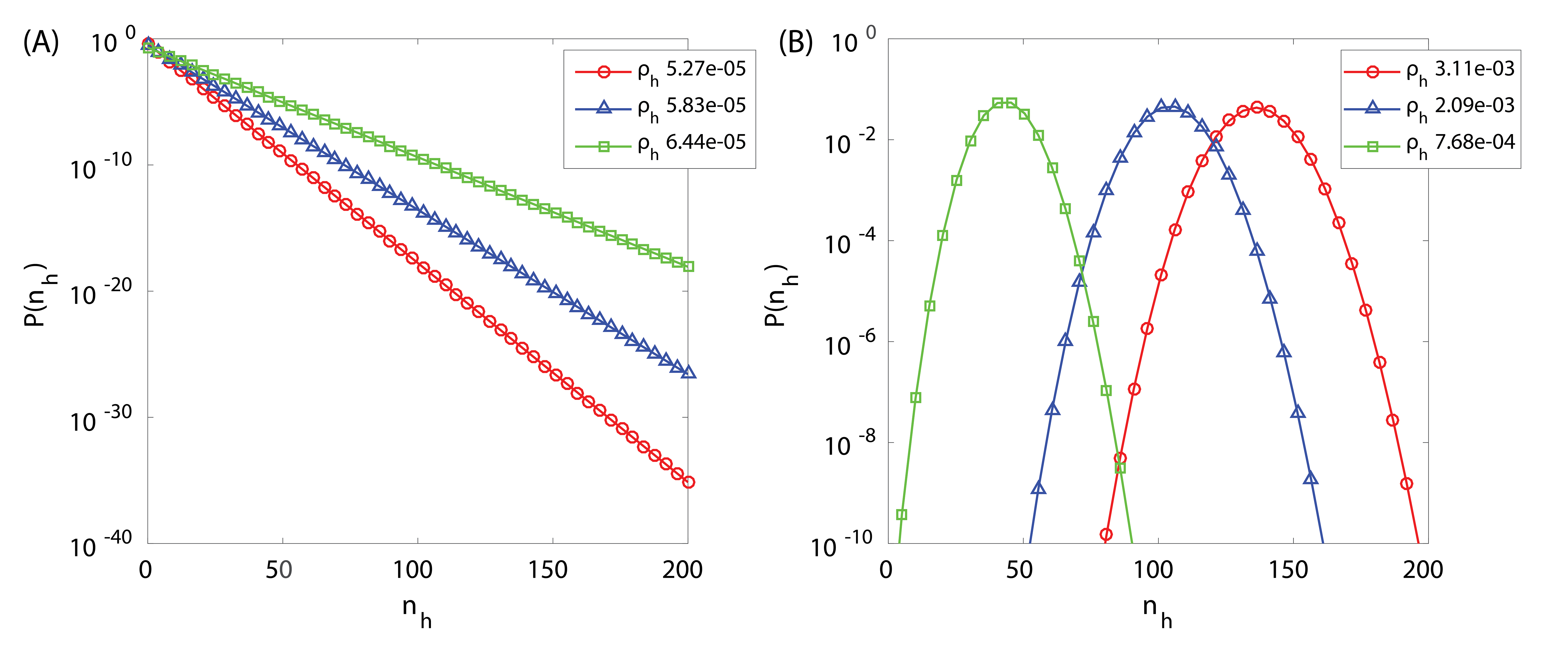

Supplement: S9 Fig — (A) Empty shells and (B) With cargo, under conditions of excess shell subunits (limiting cargo). Size distributions are obtained by solving Eq. (S2.12), with Δμc = 0.18, ΔGp = 80, and κs = 20kBT. Other parameters are from the calculations in Ref. [42] for εSC = 7.0, εCC = 1.7, and εHH = 1.8. (TIF) [file pcbi.1006351.s009.tif]

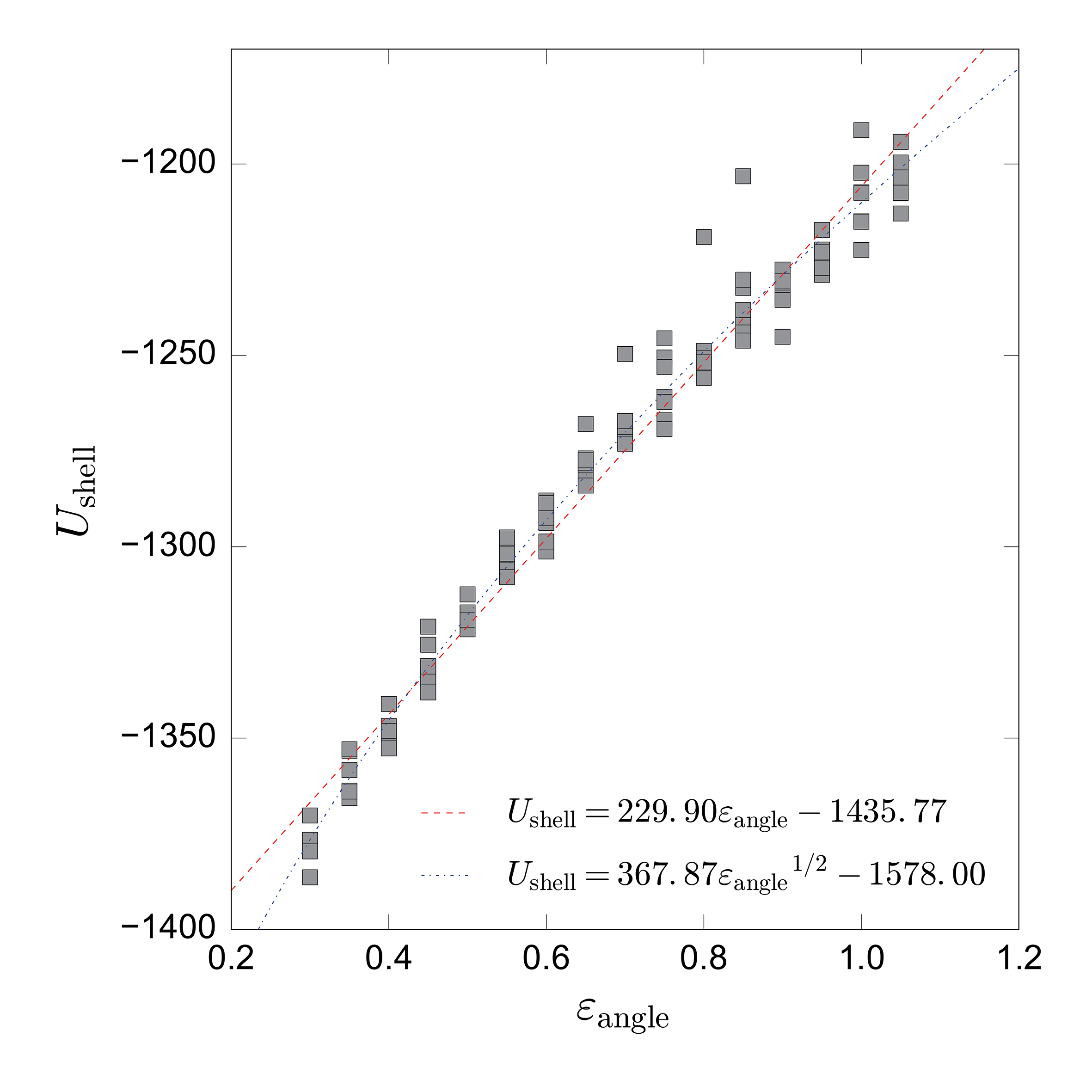

Supplement: S10 Fig — The shell has 98 hexamers and 12 pentamers, and other parameters are εHH = 1.8, εSC = 9.0, and εCC = 1.5. (TIF) [file pcbi.1006351.s010.tif]
