# Supplementary material for: The role of the encapsulated cargo in microcompartment assembly
Source: PLoS Comput Biol. 2018 Jul 31;14(7):e1006351. doi: 10.1371/journal.pcbi.1006351 (PMC6086489; doi:10.1371/journal.pcbi.1006351)
Supplement: S1 Text — (PDF) [file pcbi.1006351.s016.pdf]

S1 Model Details. Our model represents subunits as rigid bodies comprised of pseudoatoms arranged to capture the directional attractions and shape of microcompartment pentamer and hexamer oligomers. In comparison to earlier studies with patchy spheres (e.g. [A1–A3],[94]), multi-pseudoatom subunits better describe the subunit excluded volume shape [A4, A5],[48], which we find to be important for representing assembly around many-molecule cargoes. See Ref. [67] for a comparison of these approaches.

In our model, all potentials can be decomposed into pairwise interactions. Potentials involving shell subunits further decompose into pairwise interactions between their constituent building blocks – the excluders, attractors, 'Top', and 'Bottom' pseudoatoms. It is convenient to state the total energy of the system as the sum of three terms, involving shell-shell (U_{SS}), cargo-cargo (U_{CC}), and shell-cargo (U_{SC}) interactions, each summed over all pairs of the appropriate type:

$$U = \sum_{\text{shell } i} \sum_{\text{shell } j<i} U_{\mathrm{SS}} + \sum_{\text{cargo } i} \sum_{\text{cargo } j<i} U_{\mathrm{CC}} + \sum_{\text{shell } i} \sum_{\text{cargo } j} U_{\mathrm{SC}} \tag{S1.1}$$

where $\sum_{\text{shell } i} \sum_{\text{sub } j<i}$ is the sum over all distinct pairs of shell subunits in the system, $\sum_{\text{shell } i} \sum_{\text{cargo } j}$ is the sum over all shell-cargo particle pairs, etc.

Shell-shell interaction potentials. The shell-shell potential U_{SS} is the sum of the attractive interactions between complementary attractors, and geometry guiding repulsive interactions between 'Top' - 'Top', 'Bottom' - 'Bottom', and 'Top' - 'Bottom' pairs. There are no interactions between members of the same rigid body. Thus, for notational clarity, we index rigid bodies and non-rigid pseudoatoms in Roman, while the pseudoatoms comprising a particular rigid body are indexed in Greek. For subunit i we denote its attractor positions as $\{\mathbf{a}_{i\alpha}\}$ with the set comprising all attractors α, its 'Top' position $\mathbf{t}_i$, 'Bottom' position $\mathbf{b}_i$ and, for the case of subunits with no spontaneous curvature, the 'M' pseudoatom at the center of the subunit in the plane of the attractors, as $\mathbf{m}_i$.

The shell-shell interaction potential between two subunits i and j is then defined as:

$$\begin{aligned} U_{\mathrm{SS}}(\{\mathbf{a}_{i\alpha}\}, \mathbf{t}_i, \mathbf{a}_j, \mathbf{t}_j) = {} & \varepsilon_{\mathrm{angle}} \text{Ł}\left(|\mathbf{t}_i - \mathbf{t}_j|,\ \sigma_{\mathrm{t},ij}\right) \\ & + \varepsilon_{\mathrm{angle}} \text{Ł}\left(|\mathbf{b}_i - \mathbf{b}_j|,\ \sigma_{\mathrm{b}}\right) \\ & + \varepsilon_{\mathrm{angle}} \text{Ł}\left(|\mathbf{b}_i - \mathbf{t}_j|,\ \sigma_{\mathrm{tb}}\right) \mathcal{I}_{\mathrm{H}}(i) \mathcal{I}_{H}(j) \\ & + \text{Ł}\left(|\mathbf{m}_i - \mathbf{m}_j|,\ \sigma_{\mathrm{m}}\right) \mathcal{F}_{\mathrm{H}} \\ & + \sum_{\alpha,\beta}^{N_{\mathrm{a}i}, N_{\mathrm{a}j}} \varepsilon_{\mathrm{HH}} \mathcal{M}\left(|\mathbf{a}_{i\alpha} - \mathbf{a}_{j\beta}|,\ r_0, \varrho, r_{\mathrm{cut}}^{\mathrm{att}}\right) \end{aligned} \tag{S1.2}$$

The function Ł is defined as the repulsive component of the Lennard-Jones potential shifted to zero at the interaction diameter:

$$\text{Ł}(x, \sigma) \equiv \theta(\sigma - x) \left[\left(\frac{\sigma}{x}\right)^{12} - 1 \right] \tag{S1.3}$$

with $\theta(x)$ the Heaviside function. The function $\mathcal{M}$ is a Morse potential:

$$\begin{aligned} \mathcal{M}(x, r_0, \varrho, r_{\mathrm{cut}}) = {} & \theta(r_{\mathrm{cut}} - x) \times \\ & \left[\left(e^{\varrho\left(1 - \frac{x}{r_0}\right)} - 2 \right) e^{\varrho\left(1 - \frac{x}{r_0}\right)} - V_{\mathrm{shift}}(r_{\mathrm{cut}}) \right] \end{aligned} \tag{S1.4}$$

with $V_{\mathrm{shift}}(r_{\mathrm{cut}})$ the value of the (unshifted) potential at r_{cut}.

The parameter $\varepsilon_{\rm HH}$ sets the strength of the shell-shell attraction at each attractor site, $N_{{\rm a}i}$ is the number of attractor pseudoatoms in subunit i, and $\varepsilon_{\rm angle}$ scales the repulsive interactions that enforce the geometry. The function $\mathcal{I}_{\rm H}(i)$ is 1 if subunit i is a hexamer and 0 if a pentamer; thus the term $\mathcal{I}_{\rm H}(i)\mathcal{I}_{\rm H}(j)$ specifies that we only enforce Top-Bottom interactions between pairs of hexamers. We included this factor because we found that Top-Bottom interactions between hexamers and pentamers slow the process of pentamers filling in holes in hexamer shells (see the main text), and pentamer-pentamer interactions are irrelevant. The factor $\mathcal{F}_{\rm H}=0$ for subunits with T=3 preferred curvature and $\mathcal{F}_{\rm H}=1$ for subunits with zero spontaneous curvature, so that the 'M' pseudoatoms are included only for the latter case. As mentioned above, the 'M' pseudoatoms were only needed in the limit of small $\kappa_{\rm s}$, which we only considered for subunits without spontaneous curvature.

Shell-shell interaction parameter values. *Attractors:* The strength of attractive interactions is parameterized by the well-depth $\varepsilon_{\rm HH}$ for a pair of attractors on hexamers as follows. Hexamer-Hexamer edge attractor pairs (A2-A6, A3-A5, and A5-A6) have a well-depth of $\varepsilon_{\rm HH}$. Because vertex attractors (A1, A4) have multiple partners in an assembled structure, whereas edge attractors have only one, the well-depth for the vertex pairs (A1-A4 and A4-A4) is set to $0.5\varepsilon_{\rm HH}$. Similarly, for pentamer-hexamer interactions, the well-depth for edge attractor pairs (A2-A5, A3-A6) is $\varepsilon_{\rm PH}$, while the vertex interaction pairs (A1-A4 and A4-A4) have $0.5\varepsilon_{\rm PH}$. We set the ratio $\varepsilon_{\rm PH}/\varepsilon_{\rm HH}$=1.3 so that simulations without cargo form T=3 shells, or shells close in size to T=3 (see Fig. 4) for the parameter ranges we consider with cargo. Note that we cannot compare exact parameter ranges with and without cargo, since we focus on conditions for which the cargo is required for shell nucleation. Therefore, we performed our empty shell simulations with higher subunit-subunit interaction strengths, $\varepsilon_{\rm HH}=2.6$, but maintaining the ratio $\varepsilon_{\rm PH}/\varepsilon_{\rm HH}$=1.3. Interestingly, complete shells at the low stoichiometric ratio $\rho_{\rm p}/\rho_{\rm h}=0.3$ incorporated excess hexamers during assembly, but these were eventually shed resulting in complete shells with 12 pentamers and 20 hexamers.

Repulsive interactions: The 'Top' and 'Bottom' heights, or distance out of the attractor plane, are set to $h=1/2r_{\rm b}$, with $r_{\rm b}=1$ the distance between a vertex attractor and the center of the pentagon. For simulations of shells with T=3 preferred curvature, $\sigma_{\rm tb}=1.8r_{\rm b}$ is the diameter of the 'Top' - 'Bottom' interaction (this prevents subunits from binding in inverted configurations [51]), and $\sigma_{\rm b}=1.5r_{\rm b}$ is the diameter of the 'Bottom' - 'Bottom' interaction. In contrast to the latter parameters, $\sigma_{{\rm t},ij}$ the effective diameter of the 'Top' - 'Top' interaction, depends on the species of subunits i and j; denoting a pentagonal or hexagonal subunit as 'p' or 'h' respectively, $\sigma_{\rm t,pp}=2.1r_{\rm b}$, $\sigma_{\rm t,hh}=2.4r_{\rm b}$, and $\sigma_{\rm t,ph}=2.2r_{\rm b}$. The parameter r_0 is the minimum energy attractor distance, set to $0.2r_{\rm b}$, $\varrho=4r_{\rm b}$ determines the width of the attractive interaction, and $r_{\rm cut}^{\rm att}=2.0r_{\rm b}$ is the cutoff distance for the attractor potential. Since the interactions just described are sufficient to describe assembly of the shell subunits, we included no excluder-excluder interactions and $\mathcal{F}_{\rm H}$ is zero for simulations of shells with preferred curvature. For flat subunits, the diameter of the 'Top' - 'Top' interaction is equal to the diameter of 'Bottom' - 'Bottom' interaction, $\sigma_{{\rm t},hh}=\sigma_{\rm b}=2.226r_{\rm b}$, $\sigma_{\rm tb}=2.0r_{\rm b}$, and $\sigma_{\rm m}=2.026r_{\rm b}$ is the effective diameter of the middle excluders 'M'. Attractor parameters are the same as for T=3 subunits.

Cargo-cargo interactions. The interaction between cargo particles is given by

$$U_{\rm CC}(\{\mathbf{l}_i\},\{\mathbf{l}_j\}) \quad = \quad \sum_{i<j}^{N_l} \varepsilon_{\rm CC}\mathcal{L}\left(|\mathbf{l}_i-\mathbf{t}_j|\,,\ \sigma_{\rm C}, r_{\rm cut}^{\rm c}\right) \qquad \text{(S1.5)}$$

with $\mathcal{L}$ the full Lennard-Jones interaction:

$$\mathcal{L}(x,\sigma,r_{\mathrm{cut}}) = \theta(x-r_{\mathrm{cut}})\times \left\{4\left[\left(\frac{x}{\sigma}\right)^{1}2-\left(\frac{x}{\sigma}\right)^{6}\right]-V_{\mathrm{shift}}(r_{\mathrm{cut}})\right\} \tag{S1.6}$$

and $\varepsilon_{\mathrm{CC}}$ is an adjustable parameter which sets the strength of the cargo-cargo interaction, N_{l} is the number of LJ particles, the cargo diameter is $\sigma_{\mathrm{C}} = r_{\mathrm{b}}$ and the cutoff is $r^{\mathrm{c}}_{\mathrm{cut}} = 3\sigma_{\mathrm{C}}$.

Shell-cargo interactions. The shell-cargo interaction is modeled by a short-range repulsion between cargo-excluder and cargo-'Top' pairs representing the excluded volume, plus an attractive interaction between pairs of cargo particles and hexamer 'Bottom' pseudoatoms. (We do not consider pentamer-cargo attractions because there is no experimental evidence for them.) For subunit i with excluder positions $\{\mathbf{x}_{i\alpha}\}$ and 'Bottom' psuedoatom $\mathbf{b}_i$, and cargo particle j with position $\mathbf{R}_j$, the potential is:

$$U_{\mathrm{SC}}(\{\mathbf{x}_{i\alpha}\},\mathbf{R}_j) = \sum_{\alpha}^{N_{\mathrm{x}}} \text{Ł}\left(|\mathbf{x}_{i\alpha}-\mathbf{R}_j|,\sigma_{\mathrm{ex}}\right) \tag{S1.7}$$

$$+\sum_{\alpha}^{N_{\mathrm{t}}} \text{Ł}\left(|\mathbf{t}_{i\alpha}-\mathbf{R}_j|,\sigma_{\mathrm{t}}\right) \tag{S1.8}$$

$$+\sum_{\alpha}^{N_{\mathrm{b}}} \varepsilon_{\mathrm{SC}}\mathcal{M}\left(|\mathbf{c}_{i\alpha}-\mathbf{R}_j|,\ r_0,\varrho^{\mathrm{SC}},r^{\mathrm{SC}}_{\mathrm{cut}}\right)\mathcal{I}_{\mathrm{H}}(i)$$

where $\varepsilon_{\mathrm{SC}}$ parameterizes the shell-cargo interaction strength, N_{x}, N_{t}, and N_{b} are the numbers of excluders, 'Top', and 'Bottom' pseudoatoms on a shell subunit, $\sigma_{\mathrm{ex}} = 0.5r_{\mathrm{b}}$ and $\sigma_{\mathrm{t}} = 0.5r_{\mathrm{b}}$ are the effective diameters of the Excluder - cargo and 'Top' - cargo repulsions, $r_0^{\mathrm{SC}} = 0.5r_{\mathrm{b}}$ is the minimum energy attractor distance, the width parameter is $\varrho^{\mathrm{SC}} = 2.5r_{\mathrm{b}}$, and the cutoff is set to $r^{\mathrm{SC}}_{\mathrm{cut}} = 3.0r_{\mathrm{b}}$. Finally, the term $\mathcal{I}_{\mathrm{H}}(i)$ specifies that only hexamers have attractive interactions with cargo.

References

A1. Schwartz R, Shor PW, Prevelige PE, Berger B. Local Rules Simulation of the Kinetics of Virus Capsid Self-Assembly. Biophys J. 1998;75(6):2626–2636.

A2. Wilber AW, Doye JPK, Louis AA, Noya EG, Miller MA, Wong P. Reversible Self-Assembly of Patchy Particles into Monodisperse Icosahedral Clusters. J Chem Phys. 2007;127(8):085106.

A3. Baschek JE, Klein HCR, Schwarz US. Stochastic dynamics of virus capsid formation: direct versus hierarchical self-assembly. Bmc Biophysics. 2012;5. doi:10.1186/2046-1682-5-22.

A4. Nguyen HD, Reddy VS, Brooks CL. Deciphering the Kinetic Mechanism of Spontaneous Self-Assembly of Icosahedral Capsids. Nano Lett. 2007;7(2):338–344.

A5. Elrad OM, Hagan MF. Encapsulation of a polymer by an icosahedral virus. Phys Biol. 2010;7:045003.
