# Supplementary material for: The role of the encapsulated cargo in microcompartment assembly
Source: PLoS Comput Biol. 2018 Jul 31;14(7):e1006351. doi: 10.1371/journal.pcbi.1006351 (PMC6086489; doi:10.1371/journal.pcbi.1006351)
Supplement: S2 Text — (PDF) [file pcbi.1006351.s017.pdf]

. . . .

S2 Thermodynamics In this section we extend the equilibrium model of Perlmutter *et al.* [42] for shell assembly around a multi-molecule cargo to allow for formation of shells with any size. A similar approach was recently considered in Rotskoff and Geissler [58].

We consider shells composed of $n = n_\mathrm{h} + n_\mathrm{p}$ subunits, with n_h hexamer subunits and n_p pentamer subunits (or pentameric vacancies if no pentamer proteins are present). We will assume that each shell contains the minimum number of pentamers (or pentameric vacancies) dictated by topology, $n_\mathrm{p} = 12$. Based on the fact that experiments on BMCs and simulations [42,58] exhibit predominantly spherical shell geometries that are roughly but imperfectly icosahedral, we do not consider spherocylinders or other geometries [B1]), but we discuss the limits of this assumption below. Following Lidmar, Mirny, and Nelson (LMN) [B2] and Nguyen, Bruinsma, and Gelbart (NBG) [B1], we consider the elastic energy for icosahedral shells as a function of their radius of curvature R in the continuum limit, thus assuming that an icosahedral closed shell geometry is possible for any size R.

Each shell encapsulates n_c cargo molecules, given by $n_\mathrm{c} = \bar{\rho}_\mathrm{c} n^{3/2}$, with $\bar{\rho}_\mathrm{c} = \frac{a^3 \rho_\mathrm{c}}{6\sqrt{\pi}}$ and a^2 the area per shell subunit (measured at the inner surface of the shell), and ρ_c the cargo density (which we assume is approximately its liquid density). Shells assemble from a solution of free pentamers, hexamers, and cargo molecules with concentrations ρ_p, ρ_h, and ρ_c.

The total free energy density is given by

$$\begin{aligned} f_\mathrm{tot} = \sum_{\alpha=\mathrm{p,h,c}} k_\mathrm{B} T \rho_\alpha [\ln(\rho_\alpha v_0) - 1] + \\ \sum_{n=n_\mathrm{min}}^{\infty} \left(k_\mathrm{B} T \rho_n [\ln(\rho_n v_0) - 1] + \rho_n G(n) \right) \end{aligned} \tag{S2.1}$$

where the index α runs over free pentamers (p), hexamers (h), and cargo molecules (c), $v_0 = a^3$ is a standard state volume, $\rho(n)$ is the concentration of shells with n subunits, $G(n)$ is the free energy in such a shell arising from shell-shell and shell-cargo interactions, and n_min is the minimum shell size allowed by geometry (*e.g.* 12 pentamers). We then minimize f_tot with respect to $\{\rho_n\}$, subject to the constraint that the total concentrations of pentamer, hexamer, and cargo molecules $\rho_\mathrm{p}^\mathrm{T}$, $\rho_\mathrm{h}^\mathrm{T}$, and $\rho_\mathrm{c}^\mathrm{T}$ are fixed:

$$\begin{aligned} \rho_\mathrm{p}^\mathrm{T} &= \rho_\mathrm{p} + n_\mathrm{p} \sum_{n=n_\mathrm{min}}^{\infty} \rho_n \\ \rho_\mathrm{h}^\mathrm{T} &= \rho_\mathrm{h} + \sum_{n=n_\mathrm{min}}^{\infty} (n - n_\mathrm{p}) \rho_n \\ \rho_\mathrm{c}^\mathrm{T} &= \rho_\mathrm{c} + \sum_{n=n_\mathrm{min}}^{\infty} \bar{\rho}_\mathrm{c} n^{3/2} \rho_n . \end{aligned} \tag{S2.2}$$

The minimization results in the law of mass action for concentrations of shells [B3],[42]:

$$\begin{aligned} \rho(n) &= \exp\left[-\Omega(n)/k_\mathrm{B}T\right] \\ \Omega(n) &= \left(G(n) - n_\mathrm{p}\mu_\mathrm{p} - (n - n_\mathrm{p})\mu_\mathrm{h} - \bar{\rho}_\mathrm{c} n^{3/2} \mu_\mathrm{c} \right), \end{aligned} \tag{S2.3}$$

where $\Omega(n)$ is the excess free energy which includes the mixing entropy penalty associated with removing subunits and cargo particles from solution, with chemical potentials $\mu_\alpha = k_\mathrm{B}T \ln(v_0\rho_\alpha)$ for $\alpha = \{\mathrm{p}, \mathrm{h}, \mathrm{c}\}$.

We define the interaction free energy $G(n)$ as:

$$G(n) = E_\mathrm{elastic}(n) + \Delta G_\mathrm{p} + (n - n_\mathrm{p})(g_\mathrm{hh} + g_\mathrm{hc}) + \tau a^2 n + \bar{\rho}_\mathrm{c} n^{3/2} \mu_\mathrm{c}^\mathrm{liq} \,, \tag{S2.4}$$

with $\Delta G_\mathrm{p} = n_\mathrm{p}(g_\mathrm{ph} + g_\mathrm{pc})$ (provided pentamers are present) and g_ph and g_hh as the shell shell-shell binding free energy per pentamer or hexamer (we assume the shells are large enough that there are no direct pentamer-pentamer interactions), g_pc and g_hc the shell-cargo interaction free energy strengths, $\mu_\mathrm{c}^\mathrm{liq}$ the chemical potential of the cargo subunits within the packaged globule, and τ the surface tension of the cargo globule. If there are no pentamers present, then ΔG_p accounts for the 12 pentameric vacancies.

The term E_elastic gives the elastic energy of the shell arising from bending and stretching deformations, including the contributions of the 12 disclinations required by topology. In the case of a fluid membrane, the bending energy is given by the ratio of its curvature radius R to its spontaneous curvature R_0 by the Helfrich energy, $E_\mathrm{bend}(R/R_0)$, with [B4]

$$E_\mathrm{bend}(m) = 8\pi\kappa\left(1 - 2m + m^2\right) + 4\pi\kappa_\mathrm{G}, \tag{S2.5}$$

with κ and κ_G the mean and Gaussian curvature moduli.

The deformation energy for an elastic icosahedral shell without spontaneous curvature was derived by LMN [B2] and then approximately extended to include spontaneous curvature by NBG [B1]. The behavior depends on the dimensionless Föppl-von Kármán number (FvK), $\gamma = YR^2/\kappa_\mathrm{s}$ with Y the 2D Youngs modulus, which gives the relative importance of bending and stretching. Stretching energy dominates over bending when $\gamma > \gamma_\mathrm{B} \approx 130$, driving buckling of the shell [B1, B2]. Below the buckling threshold, the elastic energy is given by

$$E_\mathrm{elastic}(\gamma, \gamma_0) \approx 6\kappa B\gamma/\gamma_\mathrm{B} + E_\mathrm{bend}(\sqrt{\gamma/\gamma_0}) \qquad \text{for } \gamma < \gamma_\mathrm{B}. \tag{S2.6}$$

where $\gamma_0 = YR_0^2/\kappa_\mathrm{s}$ is the FvK for a shell at its minimum energy size ($R = R_0$), and the first term gives the energy arising from the elastic interactions between the 12 disclinations for an icosahedral structure, with $B \approx \pi/3$ a numerical constant [B2, B5]. The elastic energy from the defect interactions grows quadratically with shell size, until γ_B when it becomes favorable to screen the interaction by buckling. Above this threshold, the elastic energy in the absence of spontaneous curvature is given by [B2]

$$E_\mathrm{elastic}(\gamma, \gamma_0 = \infty) \approx 6\kappa B\left[1 + \ln(\gamma/\gamma_\mathrm{B})\right] + E_\mathrm{bend}(0) \text{ for } \gamma > \gamma_\mathrm{B}. \tag{S2.7}$$

We omit the (lengthy) expression for the case of non-zero spontaneous curvature above buckling [B1], since in the present paper we focus on the sub-buckling case for simulations with spontaneous curvature. We will consider buckling of shells with spontaneous curvature in a future work.

Mean shell size. The mean shell size can be obtained from Eqs. (S2.2) and (S2.3) as a function of the chemical potentials μ_p, μ_h, μ_c using

$$\langle n\rangle = \int n\rho(n) / \int \rho(n). \tag{S2.8}$$

Alternatively, since the total concentrations of each species $\rho_\mathrm{p}^\mathrm{T}$, $\rho_\mathrm{h}^\mathrm{T}$, and $\rho_\mathrm{c}^\mathrm{T}$ are the usual experimental control variables, it is convenient to numerically solve for the three unknown chemical potentials at fixed total concentrations.

Shell size distribution for subunits with no spontaneous curvature, in the limit of excess of hexamers. In this section we calculate the shell size distribution corresponding to the simulation results on hexamer subunits without spontaneous curvature. Based on the simulation results, we restrict the calculation to spherical cargo globules and icosahedral shells, so the complete shell contains n_{h} hexamers and 12 pentameric vacancies. We discuss the limits of this restriction below.

In the absence of cargo, and below the buckling threshold, the excess free energy in Eq. S2.3 is given by

$$\Omega(n_{\mathrm{h}}) = G_0 + \Delta\mu'_{\mathrm{h}} \tag{S2.9}$$

with $G_0 = 8\pi\kappa + \Delta G_{\mathrm{p}}$ and $\Delta\mu'_{\mathrm{h}} = \Delta\mu_{\mathrm{h}} + 6\kappa B/n_{\mathrm{B}}$ with $n_{\mathrm{B}} = 4\pi\gamma_{\mathrm{B}}\kappa_{\mathrm{s}}/Ya^2$ the threshold buckling size. Eq. (S2.9) has the same form as the free energy of a system of fluid vesicles [B6] (for simplicity we are neglecting the renormalization of κ with shell size). However, allowing for equilibrium between assembled shells and free subunits gives the form of a cylindrical micelle [B3], with an exponential shell size distribution

$$P(n_{\mathrm{h}}) = \exp\left(n_{\mathrm{h}}/\langle n_{\mathrm{h}}\rangle\right) \tag{S2.10}$$

with $\langle n_{\mathrm{h}}\rangle \approx \sqrt{\rho_{\mathrm{h}}^{\mathrm{T}} e^{\beta G_0}}$. Thus shells are polydisperse, with the standard deviation of shell sizes equal to the mean. Significant assembly requires a total subunit concentration exceeding the 'critical concentration' [B7],[94]

$$\rho^* \approx e^{\beta(g_{\mathrm{hh}} + 6\kappa B/n_{\mathrm{B}})}. \tag{S2.11}$$

As pointed out in NBG [B1], above the buckling threshold the free energy is unstable due to the presence of the log term in Eq. (S2.7), and the distribution is thus highly polydisperse.

In the presence of cargo, the excess free energy is given by

$$\Omega(n_{\mathrm{h}}) = G_0 + \Delta\mu'_{\mathrm{h}} n_{\mathrm{h}} + \bar{\rho}_{\mathrm{c}} n_{\mathrm{h}}^{3/2} \Delta\mu_{\mathrm{c}} \tag{S2.12}$$

with the chemical potential difference now including shell-cargo interactions, $\Delta\mu'_{\mathrm{h}} = g_{\mathrm{hh}} + g_{\mathrm{hc}} - \mu_{\mathrm{h}} + \tau a^2 + 6\kappa B/n_{\mathrm{B}}$, and the cargo chemical potential difference $\Delta\mu_{\mathrm{c}} = \mu_{\mathrm{c}}^{\mathrm{liq}} - \mu_{\mathrm{c}}$.

Under conditions of limiting cargo, the system will equilibrate at concentrations of free shell subunits and cargo such that $\Delta\mu'_{\mathrm{h}} < 0$ and $\Delta\mu_{\mathrm{c}} > 0$, with $\mu_{\mathrm{c}} = k_{\mathrm{B}}T\log(\rho_{\mathrm{c}} v_0)$ and $\rho_{\mathrm{c}} = \rho_{\mathrm{c}}^{\mathrm{T}} - \sum_{n_{\mathrm{h}}=n_{\mathrm{min}}}^{\infty} \bar{\rho}_{\mathrm{c}} n_{\mathrm{h}}^{3/2} \rho_{n_{\mathrm{h}}}$ accounting for the 'finite-pool' of cargo particles [B8]. The finite pool effect gives rise to a minimum in Eq. (S2.12) , and correspondingly a maximum in the shell size distribution (Fig. S9). Note that in the thermodynamic limit, the condition $\Delta\mu'_{\mathrm{h}} < 0$ should make the system unstable to other structures with a larger surface-to-area ratio, such as spherocylinders. Indeed, Cameron *et al.* [39] observed elongated structures when pentamer proteins were knocked out and RuBisCO was overexpressed. We do not allow for these in the present calculation because we do not observe them in our simulations, either because the system size is not large enough or because the initial coalescence of cargo into a spherical droplet makes these geometries kinetically inaccessible.

Determination of parameter values Comparing predictions of the equilibrium theory against BD simulation results requires a mapping between the interaction parameters of the theory (g_{hh}, g_{hc}, g_{ph}, g_{pc}, $\mu_{\mathrm{c}}^{\mathrm{liq}}$, τ, and ρ_{c}) and simulations ($\varepsilon_{\mathrm{HH}}$, $\varepsilon_{\mathrm{PH}}$, $\varepsilon_{\mathrm{SC}}$, $\varepsilon_{\mathrm{CC}}$). For this purpose, we use the mappings estimated in [42]. Note that these mappings are approximate, and we have not updated them for changes in $\varepsilon_{\mathrm{angle}}$ (and in

the case of flat subunits, the preferred subunit-subunit angle) between the two studies. Moreover, the estimates for subunit-subunit binding affinities ($g_{\mathrm{hh}}(\varepsilon_{\mathrm{HH}})$ and $g_{\mathrm{ph}}(\varepsilon_{\mathrm{PH}})$) are calculated for subunit dimerization reactions, and thus do not fully account for differences in the translational and rotational entropy of subunits within a complete shell compared to an a dimer. Thus, we can only qualitatively compare the equilibrium theory against the simulation results. However, the fitting parameters independently estimated for subunit-subunit interactions from our measurements of the shell bending modulus described next and in Fig. 6 agree reasonably well with the calculations from Ref. [42].

Estimating the shell bending modulus, κ_{s}. We tune the bending modulus in our computational model by varying the parameter $\varepsilon_{\mathrm{angle}}$. However the angular dependence of the subunit-subunit interaction arises from a combination of nonlinear repulsive and attractive potentials, and has sufficient complexity that we could not directly calculate the bending modulus. We therefore obtained rough empirical estimates of the relationship $\kappa_{\mathrm{s}}(\varepsilon_{\mathrm{angle}})$ by measuring the change in the average energy of assembled shells as a function of $\varepsilon_{\mathrm{angle}}$ and/or shell size. Note that the dependence of κ_{s} on $\varepsilon_{\mathrm{angle}}$) differs for the two versions of the model (with and without spontaneous curvature).

For shells with T=3 spontaneous curvature, we extracted a complete shell containing 98 hexamers and 12 pentamers, along with cargo, which had assembled in a simulation with parameters $\varepsilon_{\mathrm{HH}}$=1.8, $\varepsilon_{\mathrm{SC}}$=9.0 and $\varepsilon_{\mathrm{angle}}$=1. We then performed a set of BD simulations, each at a different value of $\varepsilon_{\mathrm{angle}}$ but with other parameters fixed, using the complete shell configuration as initial conditions. In each simulation we performed 10^5 time steps to allow relaxation under the new value of $\varepsilon_{\mathrm{angle}}$, followed by an additional 5×10^4 time steps during which we measured the total energy of the shell, $U_{\mathrm{shell}}(\varepsilon_{\mathrm{angle}})$, including all shell-shell attractive and repulsive interactions (but not shell-cargo interactions since these were nearly independent of $\varepsilon_{\mathrm{angle}}$). We then performed two regression analyses to fit the measured dependence of U_{shell} on $\varepsilon_{\mathrm{angle}}$ according to

$$\begin{aligned} U_{\mathrm{shell}}(\varepsilon_{\mathrm{angle}}) &= U_0 + U_{\mathrm{bend}}(\varepsilon_{\mathrm{angle}}) \\ U_{\mathrm{bend}}(\varepsilon_{\mathrm{angle}}) &= C_1 \varepsilon_{\mathrm{angle}}^p + C_2 \end{aligned} \tag{S2.13}$$

with $p = 1$ (linear regression) or $p = 1/2$ and C_1 and C_2 fit parameters.

The constant U_0 estimates the shell energy at $\varepsilon_{\mathrm{angle}} = 0$ and thus can be interpreted as the contribution from the attractive interactions and pentamers in their unperturbed configurations, $U_0 = n_{\mathrm{p}}(g_{\mathrm{ph}}) + (n - n_{\mathrm{p}})(g_{\mathrm{hh}})$. The remainder of Eq. (S2.13) captures the variation of shell energy with $\varepsilon_{\mathrm{angle}}$, and thus can be interpreted as the bending energy arising from deviations from the shell spontaneous curvature. Fig. S10 shows the fits of Eq. (S2.13) to the simulation data.

We then estimate the bending modulus from U_{bend} according to

$$U_{\mathrm{bend}}(\varepsilon_{\mathrm{angle}}) = 8\pi\kappa_{\mathrm{s}} \left(1 - \frac{n}{n_0}\right)^2 \tag{S2.14}$$

with $n = 110$ subunits in the simulated shell, and $n_0 = 32$ the number of subunits in a shell with radius equal to its spontaneous curvature R_0. For $\varepsilon_{\mathrm{angle}}$=0.5 nonlinear and linear fits result in κ_{s}=14.5 and κ_{s}=6.2 respectively. Discriminating between these fits (or any other value of p) is challenging because they primarily differ near $\varepsilon_{\mathrm{angle}} = 0$ where we are unable to obtain simulation results. Moreover, the calculated κ_{s} depends on the value obtained for U_0. Thus, we set $\kappa_{\mathrm{s}} = 10 \pm 5 k_{\mathrm{B}}T$ as an approximate average between the two fits.

Our simulations of flat subunits explore a wider range of $\varepsilon_{\mathrm{angle}}$ and shell sizes than those of simulations with spontaneous curvature. Consequently, we observed more

significant nonlinear effects, and a higher-order dependence of elastic energy on shell size than accounted for in Eq. (S2.6). Note that these nonlinearities are not consistent with the expected renormalization of bending modulus with shell size [B6], but rather arise from the very large deviations from the preferred curvature $R_0 = \infty$ for the small shells considered. Therefore, for each shell size considered in Fig. 6, we measured the interaction energy $U_{\rm shell}$ as a function of $\varepsilon_{\rm angle}$ following the procedure described above, and then estimated an effective value of $\kappa_{\rm s}$ from Eqs. (S2.13) and (S2.6) with $n/n_0 = 0$.

Our estimated bending modulus values are comparable to mechanical properties of carboxysomes measured by AFM. Using AFM nanoindentation experiments on β-carboxysomes, Faulkner *et al.* [89] estimated a 3D Young's modulus of $E = 0.6$ MPa from a linear fit or $E = 80$ MPa from a Hertzian fit to the nanoindention profiles. These estimates lie below the range of Young's modulus values measured for viruses by nanoindention, $E \in [100\text{MPa}, 2\text{GPa}]$ [B9–B12], thus suggesting that the carboxysome bending modulus lies below the range of corresponding bending modulus values for viruses, $\kappa \in [30, 600] k_{\rm B}T$.

A lower bound on the bending modulus can be estimated from the linear fit according to thin shell elasticity as [B13]

$$\kappa_{\rm s} = \frac{Eh^3}{12\,(1-\nu)^2} \tag{S2.15}$$

with h the thickness of the carboxysome shell and ν the Poisson's ratio. Using $h \approx 4.5$ nm estimated from the carboxysome structure [89] and the typical Poisson's ratio for proteins $\nu = 0.3$ [B13] results in $\kappa_{\rm s} = 1.9 k_{\rm B}T$. This is a crude estimate since the nanoindention profile is better fit by the nonlinear Hertzian model and the effective thickness h typically corresponds to the minimum thickness of the shell rather than its mean thickness; for instance the effective thickness of virus shells has been estimated at $h \approx 2$ [B13]. However, from a direct comparison of the estimated Young's modulus values for carboxysomes and viruses, it is reasonable to estimate that the carboxysome bending modulus falls in the range $\kappa_{\rm s} \in [1, 25] k_{\rm B}T$.

References

B1. Nguyen TT, Bruinsma RF, Gelbart WM. Elasticity theory and shape transitions of viral shells. Phys Rev E. 2005;72(5):051923.

B2. Lidmar J, Mirny L, Nelson DR. Virus shapes and buckling transitions in spherical shells. Phys Rev E. 2003;68(5).

B3. Safran S. Statistical Thermodynamics of Surfaces, Interfaces, and Membranes. Addison-Wesley Pub.; 1994.

B4. Helfrich W. Elastic Properties of Lipid Bilayers - Theory and Possible Experiments. Zeitschrift Fur Naturforschung C-a Journal of Biosciences. 1973;C 28(11-1):693–703.

B5. Bowick M, Nelson D, Travesset A. Interacting topological defects on frozen topographies. Phys Rev B. 2000;62(13):8738–8751. doi:10.1103/PhysRevB.62.8738.

B6. Helfrich, W . Size distributions of vesicles : the role of the effective rigidity of membranes. J Phys France. 1986;47(2):321–329. doi:10.1051/jphys:01986004702032100.

B7. van der Schoot P, Zandi R. Kinetic Theory of Virus Capsid Assembly. Phys Biol. 2007;4(4):296–304.

B8. Mohapatra L, Goode BL, Jelenkovic P, Phillips R, Kondev J. Design Principles of Length Control of Cytoskeletal Structures. Annu Rev Biophys. 2016;45(April):85–116. doi:10.1146/annurev-biophys-070915-094206.

B9. Michel JP, Ivanovska IL, Gibbons MM, Klug WS, Knobler CM, Wuite GJL, et al. Nanoindentation studies of full and empty viral capsids and the effects of capsid protein mutations on elasticity and strength. Proc Natl Acad Sci U S A. 2006;103(16):6184–6189.

B10. Roos WH, Ivanovska IL, Evilevitch A, Wuite GJL. Viral capsids: Mechanical characteristics, genome packaging and delivery mechanisms. Cell Mol Life Sci. 2007;64(12):1484–1497.

B11. May ER, Aggarwal A, Klug WS, Brooks CL. Viral Capsid Equilibrium Dynamics Reveals Nonuniform Elastic Properties. Biophys J. 2011;100(11):L59–L61.

B12. Schaap IAT, Eghiaian F, des Georges A, Veigel C. Effect of envelope proteins on the mechanical properties of influenza virus. J Biol Chem. 2012;287(49):41078–41088.

B13. May ER, Brooks CL. Determination of viral capsid elastic properties from equilibrium thermal fluctuations. Phys Rev Lett. 2011;106(18):188101–188101.
